# PM2.5/PM10 Forecasting System with Benchmarking of 44 Machine Learning Algorithms and Ensemble Learning Approaches

**DOI:** 10.3390/s26134315

**Published:** 2026-07-07

**Authors:** Pedro Mamani-Suclla, Sharon Villavicencio-Siu, Antonio Arroyo-Paz

**Affiliations:** Faculty of Engineering, Universidad Tecnológica del Perú, Arequipa 040101, Peru; u20212097@utp.edu.pe (P.M.-S.); u19302737@utp.edu.pe (S.V.-S.)

**Keywords:** air quality, PM2.5, PM10, Internet of Things (IoT), machine learning, ensemble learning, environmental prediction

## Abstract

Air pollution from particulate matter (PM2.5 and PM10) poses a serious public health risk in urban environments, particularly in areas with heavy vehicular traffic. Against this backdrop, the present study proposes an Internet of Things (IoT)-based system designed to support air quality monitoring and evidence-based decision-making regarding PM2.5 and PM10 concentrations, integrating low-cost sensors with a machine learning prediction module. The study follows an experimental-applied design with a quantitative–comparative approach. Its scientific contribution is organized around an integrated IoT-ML framework addressing a concrete gap in the literature: the lack of local empirical evidence regarding which family of machine learning algorithms delivers the greatest accuracy, stability, and computational efficiency for particulate matter forecasting in mid-altitude urban environments using low-cost sensors. On one hand, the framework proposes and deploys a four-node IoT network for continuous PM2.5 and PM10 monitoring in high-traffic urban microenvironments—representing one of the first sustained deployments with low-cost, high-temporal-resolution sensors (10-min intervals) in Arequipa, Peru. On the other hand, the study presents the most extensive benchmarking reported in the local literature: a systematic evaluation of 44 machine learning algorithms under homogeneous experimental conditions, covering classical statistical models, traditional machine learning techniques, deep learning architectures, and hybrid approaches, along with an analysis of ensemble learning strategies using Ridge stacking and K-Fold cross-validation. This unified comparative analysis—applying consistent metrics (MAE, RMSE, R^2^, and MAPE), the same prediction horizon, and a shared dataset—provides replicable empirical evidence that had not previously been reported for the urban context of Arequipa. The results show that traditional statistical models perform poorly overall, while tree-based and boosting algorithms consistently achieve R^2^ values above 0.90 for both pollutants. Ensemble models, particularly stacking with Ridge regression and cross-validation, yielded the strongest overall performance, demonstrating greater robustness and prediction stability. Explainability criteria were also incorporated, enabling an assessment of each base model’s individual contribution and identifying the variables most relevant to the prediction process. The methodological contribution provides future researchers with a rigorous reference framework for algorithm selection in environmental IoT systems. Taken together, the findings demonstrate that combining low-cost IoT networks with advanced machine learning and ensemble learning techniques constitutes an effective, scalable, and cost-efficient alternative for air quality monitoring, predictive analysis, and the support of informed mitigation strategies in urban environments.

## 1. Introduction

Air pollution caused by fine particulate matter (PM2.5) and coarse particulate matter (PM10) is among the most critical public health challenges in urban environments with high vehicular traffic [1]. Based on aerodynamic diameter, PM2.5 (≤2.5 µm) penetrates deep into the pulmonary alveoli and enters the bloodstream, contributing to cardiovascular disease, respiratory illness, diabetes, and lung cancer, thereby increasing the risk of premature mortality [2]. PM10 (≤10 µm) affects the upper respiratory tract and is associated with asthma, chronic bronchitis, and nasal irritation. The present study focuses on these two fractions for three reasons: (i) they are the most regulated pollutants under Peruvian environmental law [3]; (ii) optical scattering sensors such as the PMS5003 enable the direct measurement of both fractions [4]; and (iii) the international literature consistently identifies PM2.5 and PM10 as the indicators with the greatest health impact in high-traffic urban areas [4]. Future work could extend this framework to include NO_2_ and CO via electrochemical sensors compatible with the same IoT architecture.

In 2021, the World Health Organization (WHO) tightened its global air quality guidelines, setting annual and 24-h mean thresholds of 5 µg/m^3^ and 15 µg/m^3^ for PM2.5, and 15 µg/m^3^ and 45 µg/m^3^ for PM10, based on robust evidence linking these pollutants to cardiovascular and respiratory disease and premature mortality [2].

Peru’s Environmental Quality Standards (ECA) establish less stringent thresholds—an annual mean of 25 µg/m^3^ and a 24-h limit of 50 µg/m^3^ for PM2.5, and 50 µg/m^3^ and 100 µg/m^3^ for PM10, respectively [3]—yet these limits remain difficult to meet consistently in cities experiencing rapid vehicle fleet growth and insufficient urban road planning [4].

Arequipa presents a particularly complex monitoring challenge that conventional approaches have struggled to address. As Peru’s second-largest city, it combines a rapidly expanding vehicle fleet and high-traffic corridors with a distinctive set of topographic, meteorological, and socioeconomic conditions that intensify pollutant accumulation [5]. The city sits at approximately 2300 m above sea level in a high-altitude arid basin surrounded by mountains, a geography that constrains atmospheric dispersion and promotes the formation of thermal inversions—conditions under which pollutants become trapped near the surface for extended periods. Reduced atmospheric pressure at this altitude further affects combustion efficiency in older vehicles, amplifying emission levels relative to lower-altitude cities. These factors are compounded by a vehicle fleet dominated by aging, poorly maintained units and limited access to emissions inspection and enforcement infrastructure—socioeconomic realities that increase per-vehicle emission loads. Recent studies conducted in different districts of the city confirm that PM10 levels frequently and substantially exceed both national ECAs and WHO guidelines [1], while other research documents significant relationships between local atmospheric vertical structure and PM2.5/PM10 concentrations, with some sectors surpassing annual ECA thresholds during coincident pollution episodes [6]. Together, these conditions make Arequipa a high-priority case for spatially and temporally resolved air quality monitoring—yet existing fixed-station networks offer limited spatial coverage and are unable to capture the fine-grained variability across intersections, avenues, and urban microenvironments that is necessary for evidence-based mobility policy and early warning systems targeting vulnerable populations.

Against this backdrop, Internet of Things (IoT)-based monitoring systems using low-cost sensors have emerged as a promising complement to traditional networks. These systems enable real-time measurement of PM2.5, PM10, and meteorological variables, transmitting data to web dashboards and supporting flexible deployment in priority áreas [7]. Their scalability makes them especially valuable in contexts—such as Arequipa—where economic and infrastructural constraints limit the feasibility of dense fixed-station networks [8].

Simultaneously, data-driven methods have advanced considerably in air quality modeling. Recent reviews indicate that machine learning and deep learning models—including Random Forest (RF), XGBoost, artificial neural networks, LSTM, CNN, and hybrid architectures—can achieve high predictive accuracy for PM2.5 and PM10 [9,10]. Ensemble and hybrid approaches that combine multiple models have demonstrated superior forecasting performance at both hourly and daily scales by exploiting the complementary strengths of individual algorithms [11]. However, the literature consistently shows that no single model generalizes across cities, seasons, and forecasting horizons; relative model performance is highly sensitive to local environmental conditions, seasonality, and input variable selection [12]. This underscores the necessity of city-specific benchmarks before deploying operational solutions.

Existing comparative studies in air quality prediction have typically evaluated a limited subset of algorithms—most commonly between three and ten models—under heterogeneous experimental conditions that prevent direct cross-study comparison [12]. Furthermore, the majority of these benchmarks have been conducted in large metropolitan areas at low altitudes with access to regulatory-grade reference stations, leaving mid-altitude cities with constrained monitoring infrastructure significantly underrepresented in the literature. Critically, no prior study has simultaneously integrated a locally deployed low-cost IoT network with a systematic, reproducible evaluation of 44 algorithms spanning five model families—statistical, linear, tree-based, deep learning, and hybrid—under identical conditions and using a shared dataset collected at high temporal resolution. This scale of benchmarking is not merely quantitative: by covering the full spectrum from classical baselines to state-of-the-art ensemble architectures within a single experimental framework, the present study enables a type of cross-family comparison that isolated evaluations cannot provide. Moreover, the integration of ensemble learning strategies and explainability criteria within the same framework—applied directly to locally collected IoT data—represents a methodological contribution that extends beyond algorithm selection toward a replicable decision-support tool adaptable to other constrained urban environments.

This is precisely the gap that motivates the present study. Mid-altitude cities such as Arequipa lack continuous, low-cost monitoring systems that systematically integrate IoT technology with a rigorous comparative evaluation of predictive models—a gap that prevents the accumulation of local empirical evidence regarding which machine learning algorithm families perform best under these specific environmental conditions. Studies that combine IoT monitoring networks with a broad, systematic, and reproducible algorithm comparison remain scarce in the regional literature, as do training and validation frameworks capable of identifying the factors driving PM2.5 and PM10 peak episodes.

The present research addresses this gap through an experimental-applied, quantitative, descriptive-comparative design. The experimental component consists of deploying a real IoT network to collect primary data; the applied component involves implementing and evaluating predictive models on those data; and the comparative component entails the systematic benchmarking of 44 algorithms under homogeneous conditions. Specifically, the proposed integrated IoT-ML architecture comprises (i) a low-cost four-node monitoring network deployed in high-traffic urban microenvironments; (ii) a data preprocessing and quality control pipeline; (iii) an exhaustive, reproducible benchmarking of 44 algorithms across five categories; and (iv) an ensemble learning strategy combining Ridge stacking with temporal cross-validation. This framework constitutes the core methodological contribution of the study and is designed to be transferable to other urban contexts facing similar constraints.

The overall objective is to design and validate this framework for PM2.5 and PM10 prediction in the local Arequipa context through systematic benchmarking of 44 machine learning models and ensemble learning methods. Three specific objectives guide the work: (SO1) Deploy a four-node low-cost IoT network for continuous measurement of PM2.5, PM10, and meteorological variables at 10-min intervals in high-traffic areas of the Socabaya district, Arequipa. (SO2) Conduct an exhaustive and reproducible benchmark of 44 algorithms spanning statistical, linear, tree-based, deep learning, and hybrid model families under identical experimental conditions, using MAE, RMSE, R^2^, and MAPE as evaluation metrics. (SO3) Construct and evaluate ensemble learning strategies using Ridge stacking with temporal cross-validation.

## 2. State of the Art

Air quality research has evolved along two main lines: localized, high-resolution monitoring through low-cost sensors and IoT devices, and AI-driven prediction models capable of processing large volumes of environmental data. When combined, these approaches significantly improve the spatial and temporal resolution of particulate matter measurements (PM2.5/PM10), particularly in urban corridors where vehicular traffic introduces considerable concentration variability.

### 2.1. Low-Cost Sensor Air Quality Monitoring

Traditional monitoring stations deliver high-precision measurements but come at a considerable cost, which limits their spatial coverage. This has driven growing interest in low-cost sensor networks integrated with IoT platforms, which enable dense, continuous observations across wider areas. Recent literature highlights three key advantages of these networks: scalability, spatial granularity, and ease of deployment in urban environments. At the same time, researchers consistently flag two persistent challenges: sensor calibration and data quality control [13].

Reviews of IoT-based environmental monitoring systems suggest that the most widely adopted architecture combines sensing nodes with WiFi, LoRaWAN, or 4G connectivity, along with cloud storage and data visualization layers. However, two critical aspects are often overlooked in practice: data traceability and data integrity—the latter encompassing outlier detection, packet loss, and sensor drift [14]. In this context, studies documenting the full implementation of IoT-based monitoring and prediction pipelines underscore the operational value of end-to-end workflows that integrate data acquisition, transmission, preprocessing, modeling, and visualization into a single coherent process [15].

### 2.2. IoT Architecture and Connectivity

The growing adoption of IoT-based air quality solutions has accelerated the shift toward hybrid edge-cloud architectures, where edge computing reduces latency and performs local data filtering, while cloud infrastructure supports dashboards and advanced analytics. Processing data at the edge avoids transmitting raw, unfiltered data streams to the cloud, resulting in substantially lower bandwidth consumption and making the system more cost-efficient as it scales [16].

Complementing edge–cloud designs, several proposals integrate IoT with machine learning to enable real-time forecasting, where models are continuously updated as new sensor data arrives. These systems prioritize three key operational factors: service availability, efficient data stream management, and real-time visualization capabilities [17]. Regarding connectivity, LoRaWAN has emerged as a preferred option for outdoor air quality monitoring due to its long range and low power consumption. Field deployments demonstrate its effectiveness in covering large geographic areas with minimal infrastructure, though its limited data transmission rate requires careful attention to sampling strategies and data packaging techniques [18].

### 2.3. Calibration, Quality Control and Data Reliability

Calibration is a critical concern in low-cost sensor networks, as even minor deviations can compromise both monitoring accuracy and predictive performance. Machine learning has become the dominant approach for quality control in this context. The literature identifies three broad categories of methods: classical models such as regression, Random Forest, and SVM; deep learning architectures including CNN, LSTM, and autoencoder networks; and hybrid approaches combining both. A consistent finding across studies is that more complex models tend to yield better predictive accuracy, but at the cost of higher data requirements and reduced interpretability [18].

More targeted calibration studies show that accounting for environmental variability—particularly fluctuations in temperature and humidity—leads to meaningful improvements in sensor correction performance [19]. Taken together, the evidence makes clear that a functional IoT network cannot be limited to raw data acquisition. It must incorporate a structured pipeline for data validation, cleaning, and calibration to ensure that measurements are reliable enough for both scientific analysis and real-world applications [20].

### 2.4. PM2.5, PM10 Prediction

Predictive modeling of particulate matter concentrations has advanced considerably in recent years. Although time series methods and traditional regression models laid the groundwork for early air quality forecasting, current research increasingly favors machine learning and deep learning techniques, which are better suited to capture the complex, nonlinear relationships between pollutants and meteorological variables. Systematic reviews confirm that models such as Random Forest, XGBoost, SVM, neural networks, and recurrent architectures can achieve strong predictive performance when trained on locally representative data and subjected to rigorous validation [13].

Studies focused specifically on PM2.5 prediction frequently report the use of LSTM, CNN, GRU, and various hybrid architectures. However, drawing direct comparisons across these studies remains difficult, as they differ substantially in their input variables, forecasting horizons, spatial scales, and evaluation metrics [21]. This lack of standardization has motivated the development of ensemble and hybrid solutions—such as combining Random Forest with LSTM—designed to leverage the complementary strengths of multiple modeling approaches. The incorporation of meteorological variables alongside machine learning or deep learning algorithms has been shown to consistently improve forecast robustness [22].

Furthermore, evidence across studies suggests that model performance is shaped by contextual factors including seasonal patterns, local emission profiles, and the selection of predictor variables. This reinforces the widely held view that no universal modeling solution exists, and that predictive frameworks must be tailored to the specific characteristics of each study area [23].

### 2.5. On-the-Fly, Mobile Strategies and City Still Traffic Situations

Beyond fixed monitoring stations, mobile and vehicle-based approaches have gained traction as complementary methods for capturing spatial variability in air pollution. Vehicle-mounted monitoring systems are particularly valuable for estimating population exposure during commuting and daily mobility, enabling data-driven decisions about alternative routes with lower pollution levels [24]. Similarly, mobile monitoring campaigns—where measurements are collected across multiple locations—have proven effective for fine-scale pollution mapping. These high-resolution spatial datasets can support the identification of lower-exposure routes and inform targeted interventions aimed at protecting vulnerable population groups [25].

### 2.6. Local Evidence and the Need for Specific Studies in Arequipa

Arequipa experiences recurring pollution episodes closely tied to the atmospheric dynamics of the region. Local studies have examined the relationship between atmospheric vertical profiles and particulate matter concentrations, finding that annual regulatory limits are exceeded in certain areas [6]. Measurements of PM10 have further revealed considerable spatial variability, with concentration levels diverging significantly from established standards across different zones of the city [1]. These findings highlight the need for enhanced monitoring with greater spatial and temporal resolution, particularly in high-traffic corridors where pollutant concentrations tend to be most elevated, see Table 1.

### 2.7. Identified Gaps (GAP)

Despite the proliferation of IoT-based air quality monitoring systems, few studies report sustained deployments in urban microenvironmental settings underpinned by clear, standardized methodological protocols. A recurring limitation in works employing low-cost sensors is inconsistent data quality, which introduces uncertainty that propagates through subsequent analysis and modeling stages. In this regard, systematic and large-scale evaluations of multiple machine learning algorithms under real operating conditions remain scarce, as do reproducible comparisons across a sufficiently diverse set of modeling approaches.

Moreover, although predictive model performance is widely acknowledged to be highly context-dependent, few studies have conducted exhaustive benchmarking of a broad range of machine learning algorithms using consistent evaluation metrics and uniform prediction horizons. This gap makes it difficult to draw unbiased, repeatable comparisons between methods. In particular, ensemble strategies—including stacking and other multi-model combinations—have received only partial attention in the literature, often tested with a limited number of base models, despite their demonstrated potential to improve predictive accuracy, stability, and robustness in the face of the inherent variability of environmental data.

Finally, at the local scale, the absence of hyperlocal databases with high spatial resolution and dynamic updating capacity severely constrains the feasibility of consistent predictive analyses and systematic algorithm benchmarking in real urban environments. This deficit has curtailed the development of comparative studies capable of evaluating, under locally specific conditions, the relative performance of multiple predictive models and ensemble strategies applied to PM2.5 and PM10 forecasting.

## 3. Methodology

**Figure 1:**
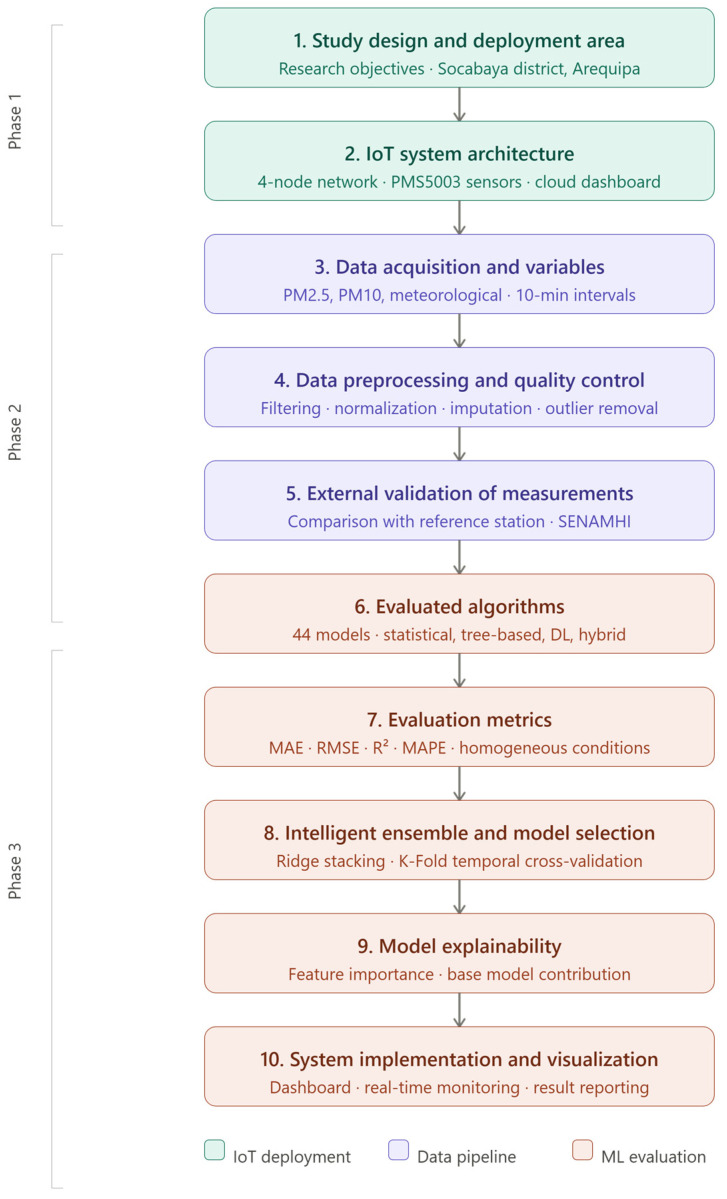
Diagram of the methodological framework.

The methodology integrates IoT-based environmental monitoring with predictive modeling, supported by systematic data validation, rigorous model evaluation, and explainability analysis to ensure the reliability and interpretability of the results, see Figure 1:

### 3.1. Field of Test and Study Design

This study was conducted within an applied experimental framework, in which field data were collected and subsequently used to develop time-based predictive models. The methodological objective was twofold: first, to deploy a low-cost IoT monitoring network capable of measuring particulate matter concentrations (PM1, PM2.5, and PM10) alongside temperature and humidity at regular time intervals in an urban area characterized by high motor vehicle traffic; and second, to design and implement forecasting models capable of predicting future PM concentrations at defined time horizons within the same setting.

The deployment was carried out in the district of Socabaya, Arequipa, Peru, using four IoT nodes positioned at traffic-representative locations. Placement sites were selected based on their urban exposure and microenvironmental representativeness, encompassing major avenues, intersections, and areas subject to recurrent vehicular congestion, see Table 2.

The four IoT nodes were distributed according to a stratified sampling strategy based on roadway type and traffic density, prioritizing the representativeness of distinct urban microenvironments. Node N1 was placed at the main square of Socabaya, an area characterized by low vehicle speeds and mixed pedestrian–vehicular flow, typical of local commercial zones with moderate traffic activity. Node N2 was installed on Av. Salaverry, an arterial road with sustained bidirectional vehicle flow throughout most of the day. Node N3 was positioned on Av. Socabaya, a major trunk road connecting key districts of the area, marked by high traffic demand and recurrent vehicular congestion. Finally, node N4 was located in the Socabaya prison sector, an area with a notable concentration of heavy transport vehicles. Collectively, this spatial distribution captures the dominant exposure microenvironments experienced by the local population: slow mixed traffic, sustained arterial flow, and heavy freight circulation.

**Figure 2:**
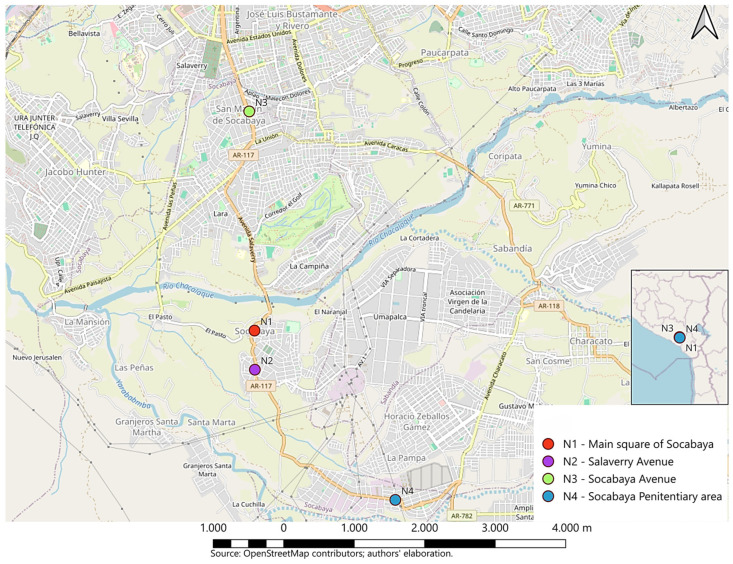
Map of the study area showing the spatial distribution of the four IoT monitoring nodes.

Regarding the generalizability of the findings, it is important to note that the results are specific to the context of Arequipa—a city situated at approximately 2335 m above sea level, characterized by low relative humidity and a topographic basin that promotes the accumulation of pollutants. Nevertheless, the proposed methodological framework—which integrates incremental IoT network deployment, systematic algorithm benchmarking, and ensemble modeling—is transferable to other urban settings, requiring only the reconfiguration of node placement to reflect local urban and environmental conditions. Future work should consider expanding the monitoring network to additional districts within the Arequipa metropolitan area, such as Cayma, Cerro Colorado, and José Luis Bustamante y Rivero, in order to characterize the spatial heterogeneity of PM2.5 and PM10 at the metropolitan scale, see Figure 2.

### 3.2. IoT System Architecture

**Figure 3:**
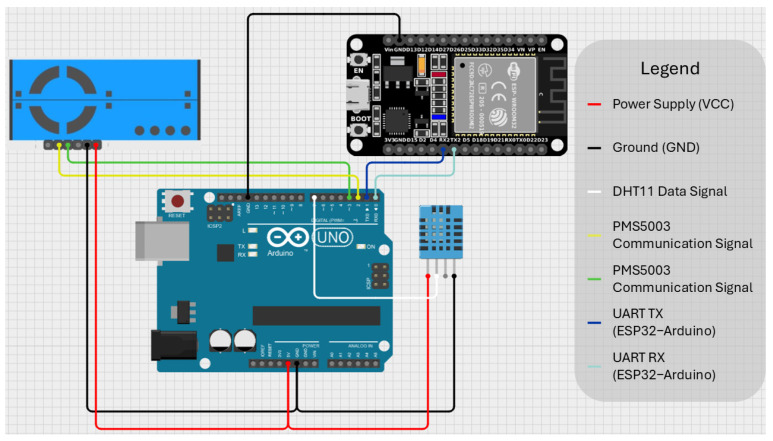
IoT system architecture diagram. Red lines indicate the power connections (Vcc), black lines indicate ground (GND), and the yellow, green, and blue lines indicate the signal/data connections between the Arduino UNO, the ESP32 module, the particulate-matter sensor, and the DHT temperature and humidity sensor.

**Figure 4:**
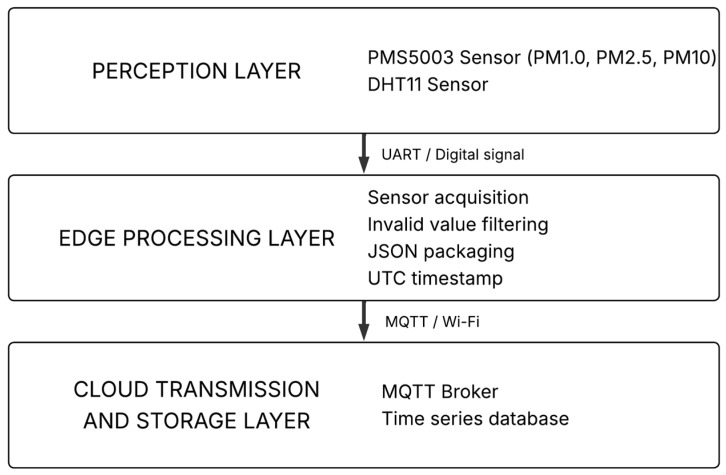
IoT system architecture structure layer diagram.

The proposed system adopts a hybrid edge-cloud architecture in which each IoT node is responsible for local data acquisition and preliminary packaging at the network edge, before transmitting the processed data to a cloud storage layer where it can be visualized and further analyzed. This design is consistent with approaches reported in the broader IoT-based environmental monitoring literature, where edge-level processing ensures continuous system operation, while cloud infrastructure provides scalability, long-term data storage, and support for more computationally demanding analyses, see Table 3 and Figure 3 and Figure 4.

**Figure 5:**
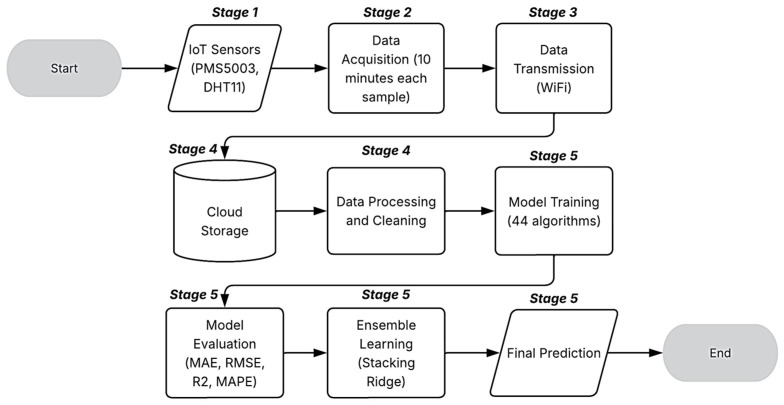
Chain of the suggested system that would assist in monitoring and forecasting PM 2.5 and PM10 using IoT sensor and machine learning models.

Each node integrates:

The system is structured around a hybrid edge-cloud platform organized into three interconnected layers that work in concert to ensure reliable environmental data acquisition, processing, and storage:

1. Perception layer: The PMS5003 sensor measures PM1.0, PM2.5, and PM10 concentrations via laser optical scattering (wavelength: 650 nm), with a measurement range of 0–500 µg/m^3^ and an accuracy of ±10% for PM2.5. Communication with the microcontroller is established via the UART protocol at 9600 baud. The DHT11 sensor records temperature over a range of −20 to 50 °C with a precision of ±2 °C, and relative humidity from 0 to 100% with a resolution of ±5% RH. Both sensors were selected based on their favorable cost-to-performance ratio, low power consumption, and straightforward integration with open-source IoT platforms.
2. Edge processing layer (Edge): Each node is built around an ESP32 microcontroller, which serves as the central processing unit, featuring 520 KB of SRAM and Wi-Fi connectivity (802.11 b/g/n). The on-board firmware handles sensor data acquisition, packaging each reading as a JSON object with a UTC timestamp, and applies a first-stage filter to discard physically implausible values—specifically, PM readings below 0 or above 500 µg/m^3^. This edge-level preprocessing reduces server load and ensures that only valid measurements are forwarded to the cloud layer.
3. Transmission and cloud storage layer: Data are transmitted at 10-min intervals to a cloud broker via the MQTT protocol. MQTT was selected for its low bandwidth requirements (overhead < 2 KB per message), tolerance to intermittent connectivity, and compatibility with low-power IoT devices. At the broker, incoming data are stored in a time series database and made accessible through a web dashboard that enables near real-time visualization with an average latency of under 5 s, see Figure 5.

This architecture ensures full traceability of every record from sensor to storage, supporting the reproducibility of the study. The end-to-end operational flow can be summarized in five sequential steps:

- (E1) Data sensors automatically capture data every 10 min.
- (E2) Preprocessing/Packaging along the edge by ESP32.
- (E3) MQTT/Wi-Fi communication to a cloud server.
- (E4) Storage, cleaning and data quality control.
- (E5) Predictive modeling using the 44 algorithms and building up the final ensemble.

### 3.3. Information Collection and Variables That Were Put into Consideration

Data collection was carried out over an extended continuous period spanning approximately 180 days. Throughout this period, the four deployed IoT nodes simultaneously recorded the following variables at 10-min intervals: PM1.0 concentration (µg/m^3^), PM2.5 concentration (µg/m^3^), PM10 concentration (µg/m^3^), ambient temperature (°C), and relative humidity (%).

Each stored record contains three key elements: a unique node identifier (N1, N2, N3, and N4), a timestamp in ISO 8601 format (UTC−5, Peru Standard Time), and the five corresponding measured values. This structure ensures full traceability of the origin of each measurement.

**Figure 6:**
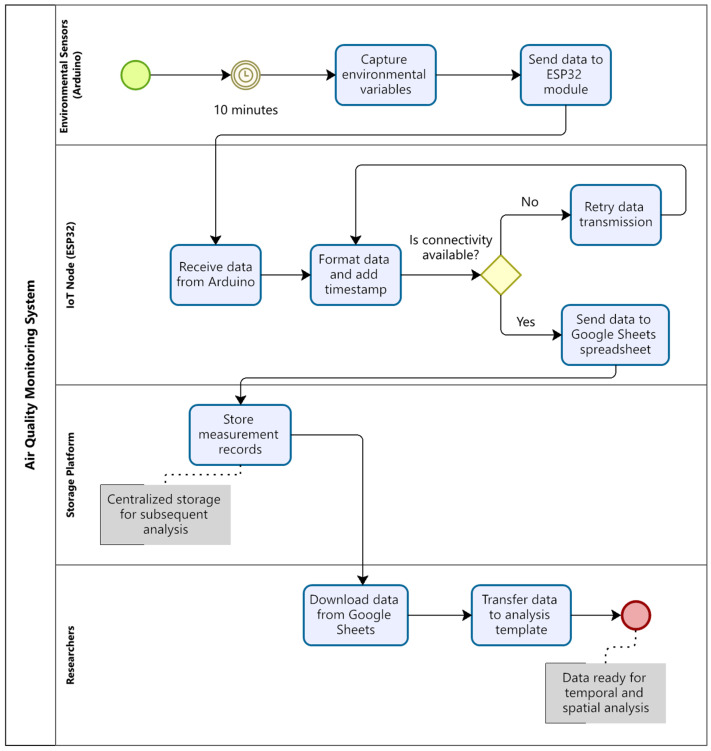
Data acquisition workflow diagram. The green circle marks the start of the process and the red circle its end; blue rounded rectangles represent tasks, the yellow diamond represents a decision point (connectivity check), and the clock symbol denotes the 10-min sampling interval. Solid arrows indicate the process sequence flow, whereas dotted lines indicate associations with the centralized data storage.

The 10-min sampling frequency yields 144 records per node per day, resulting in 576 records per day across the full four-node network. At the end of the monitoring period, the final dataset comprises 25,920 valid records, stored in CSV format with UTF-8 encoding to facilitate processing and analysis, see Figure 6.

A data quality control layer was implemented as a foundational step to ensure the reliability of the subsequent predictive analysis. During this process, 2.3% of records were removed due to anomalous values, specifically those with PM concentrations outside the physically plausible range (below 0 or above 500 µg/m^3^) and those with duplicated timestamps.

Missing data were handled through a differentiated protocol based on gap duration. Gaps of up to 30 min—equivalent to three consecutive missing readings—were filled using linear interpolation. Gaps exceeding 30 min were flagged and excluded from the analysis, as interpolating over extended periods risks introducing significant bias and obscuring genuine air quality variation events.

This procedure is essential to ensure the reproducibility of the study and to provide other researchers with a transparent account of the origin, quality, and limitations of the data used to train the predictive models.

### 3.4. Data Quality Control and Preprocessing

Sensor data are inherently susceptible to noise, outliers, and time-induced errors, making preprocessing and quality control an essential step prior to predictive model development. The analysis was performed on a dataset of 25,920 records encompassing measurements of particulate matter (PM2.5 and PM10), meteorological variables (temperature and relative humidity), and temporal identifiers (date and time). The preprocessing pipeline was designed to ensure temporal coherence, statistical consistency, and signal stability across the time series, and comprised the following stages:

1. Temporal normalization was performed by merging the date and time fields into a single, unified timestamp, ensuring chronological alignment across all records. Sampling continuity was then verified by confirming that measurements were consistently spaced at 10-min intervals, in accordance with the system configuration.
2. A descriptive statistical analysis was conducted as part of the quality control procedure, examining key distributional parameters including the mean, median, standard deviation, variance, and interquartile values. In addition, all measurements were verified to fall within physically plausible ranges, consistent with the expected operational limits of each variable.
3. Weekly and monthly aggregate statistics were computed to assess the temporal stability of the data. The coefficient of variation was calculated as an internal measure of signal variability, providing an indicator of the degree of noise present in the recorded signals.

**Figure 7:**
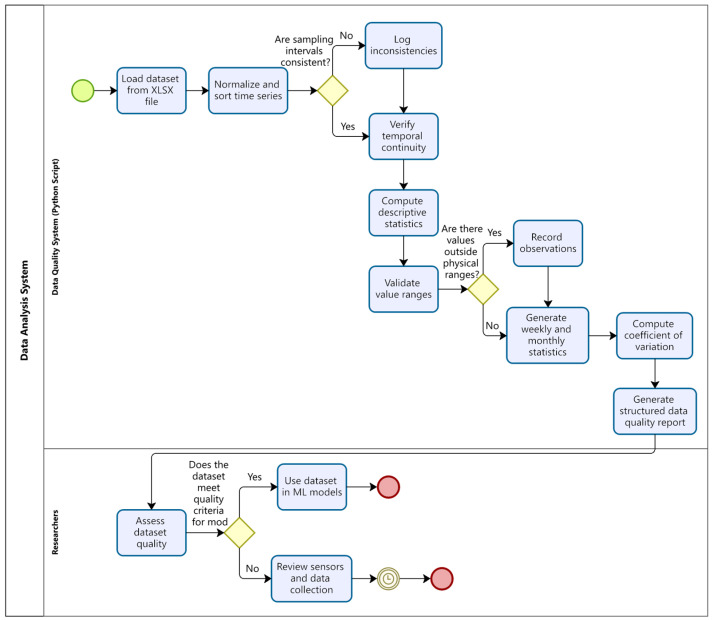
IoT data preprocessing and quality control workflow. The green circle marks the start of the process and the red circles mark its end points; blue rounded rectangles represent tasks, the yellow diamonds represent decision points (gateways), and the clock symbol denotes a timed/wait event. Arrows indicate the process sequence flow, and the Yes/No labels show the outcome of each decision.

Literature consistently identifies data quality assurance as a critical component of IoT-based environmental monitoring networks, given that errors at this stage propagate directly into predictive model outputs [20]. Accordingly, the implemented preprocessing procedure prioritized time series coherence and consistency as a prerequisite for reliable machine learning model training, see Figure 7.

### 3.5. Externally Validated Measurements

The reliability of measurements obtained from low-cost optical scattering sensors such as the PMS5003 is subject to several well-documented sources of uncertainty that must be explicitly addressed. These include (i) sensitivity to ambient relative humidity, which can cause the hygroscopic growth of particles and lead to the overestimation of PM concentrations when relative humidity exceeds 70%; (ii) sensor drift over extended deployment periods, as optical components degrade and baseline readings shift progressively; and (iii) particle composition and size distribution variability, since the PMS5003 is factory-calibrated using a specific particle type (PSL spheres) that may not reflect the optical properties of locally dominant aerosols in Arequipa’s high-altitude arid environment.

To address these limitations, a multi-stage validation and quality assurance protocol was implemented. First, prior to field deployment, all four PMS5003 units were cross-calibrated against each other under identical controlled indoor conditions during a 72-h co-location period. Units exhibiting systematic deviations greater than ±15% relative to the median of the group were recalibrated by adjusting node-level correction factors. This inter-unit consistency check is consistent with established protocols for low-cost sensor networks [18]. Second, temperature and humidity readings from DHT11 sensors were corrected using manufacturer-supplied calibration curves, and all PM readings were post-processed with a humidity correction factor following the approach proposed in the literature for optical sensors [19]. Third, a firmware-level range filter was applied at the edge layer to reject physically implausible readings (PM < 0 or PM > 500 µg/m^3^) before transmission.

Regarding external validation, the IoT prototype measurements were compared against publicly available air quality data from the IQAir platform and against Peru’s Environmental Quality Standards (ECA) established by MINAM. Table 4 presents mean values and ranges for both PM2.5 and PM10. The average concentrations recorded by the prototype are consistent with expected levels for urban areas in Arequipa and remain below national regulatory thresholds. It is important to note that IQAir data represent daily aggregates derived from a combination of satellite estimates and ground stations, and cannot substitute for calibration against co-located certified reference equipment. This comparison is therefore included solely as a contextual plausibility check—to verify that the order of magnitude and temporal trends of IoT measurements are coherent with independently available information—and not as a formal calibration procedure, see Table 4.

To further evaluate the generalizability of the modeling pipeline, an external validation at the predictive modeling level was conducted using an independent air quality dataset from Mexico City, sourced from the Mendeley Data repository. This dataset provides hourly PM2.5, PM10, and meteorological measurements under different environmental conditions, enabling an assessment of whether the processing and modeling pipeline produces consistent results beyond the local Arequipa context. The comparison maintained identical experimental conditions across both datasets, including the same preprocessing pipeline, time variable generation, evaluation metrics (MAE, RMSE, R^2^, and MAPE), training/test partition scheme, and base models. A representative subset of algorithms spanning all model families was evaluated: Ridge (linear), SVR with RBF kernel (non-linear), XGBoost (tree-based), and LSTM (deep learning). Ensemble strategies—including weighted averaging and Ridge stacking with K-Fold cross-validation—were also assessed to determine whether performance improvements observed locally were reproducible under different environmental conditions.

It is acknowledged that the absence of a co-located certified reference monitor (such as a GRIMM or TSI optical particle counter operating under regulatory conditions) represents a limitation of the present study. Future deployments should incorporate formal co-location calibration against reference-grade equipment to produce fully traceable measurement uncertainty estimates in accordance with international metrological standards.

### 3.6. Evaluated Algorithms

A total of 44 algorithms, including both machine learning and deep learning algorithms, were evaluated to ascertain their predictive performance. The selection of these algorithms was not an arbitrary decision; rather, it was the result of a methodical review of literature published between 2018 and 2025 on PM2.5 and PM10 prediction in urban environments. This review identified the most frequently reported types and algorithms in studies on air quality [9,21,23].

In order to ensure the comparative validity of the benchmarking and to eliminate possible experimental biases, all of the algorithms were estimated using strictly homogeneous conditions. The dataset under consideration contains 25,920 records and has been organized according to a chronological ordering. The dataset includes the following variables: PM 2.5, PM 10, temperature, humidity, and derived time. The dataset is partitioned into three segments, each comprising 70%, 15%, and 15% of the total records, respectively. The segments are designated for training, validation, and final evaluation, respectively. The temporal ordering of the series is preserved within each segment. The assessment measures employed include MAE, RMSE, R, and MAPE, calculated on the final evaluation set.

The inclusion of a broad spectrum of algorithms—including models expected to underperform under certain conditions—was intentional and methodologically motivated. The objective of the benchmark was twofold: first, to identify the most effective predictor, and second, to systematically evaluate how different modeling assumptions behave under the specific characteristics of PM2.5 and PM10 time series collected from low-cost IoT sensors in a mid-altitude urban environment.

Consequently, the benchmark incorporated the following elements:

- The establishment of minimum predictive reference levels is predicated on simple statistical baselines.
- The utilization of interpretable linear models is imperative for the evaluation of linear dependencies.
- Nonlinear tree-based and kernel-based methods have been developed for the purpose of capturing complex interactions.
- Deep learning architectures have been demonstrated to excel in the specialized field of temporal dependency modeling.
- Hybrid and ensemble approaches have been developed to leverage complementary predictive strengths.

It was hypothesized that certain statistical models, including multiplicative Holt–Winters, would demonstrate sensitivity to abrupt variability and non-multiplicative seasonal behavior. Nevertheless, their inclusion provides valuable scientific evidence regarding the limitations of classical forecasting assumptions in highly dynamic urban pollution datasets. Consequently, poor-performing models were retained in the analysis to preserve benchmarking completeness, avoid selection bias, and support transparent cross-family comparison under identical experimental conditions.

The algorithms were divided into five categories, each of which had a specific objective in the comparative analysis:

- (C1) Statistical reference models (baseline): This category encompasses a range of methods, including Naive, Seasonal Naive, Moving Average, Simple Exponential Smoothing (SES), Holt, Holt–Winters Additive and Multiplicative, AR, MA, ARMA, ARIMA, SARIMA, and SARIMAX. The advanced technique is distinguished by its unique cornerstones of metrics, which serve as the primary means of evaluating its superiority over conventional statistical models.
- (C2) Linear regularized models: This is the category of Linear regression, Ridge, Lasso, and Elastic Net. These models represent the two extremes of the spectrum of low-cost computation and high interpretability, which are fundamental characteristics in any comprehensive comparison.
- (C3) Tree-based and boosting: This category includes k-Nearest Neighbors (kNN), Regression Tree, Random Forest, Extra Trees, Gradient Boosting, XGBoost, LightGBM, CatBoost, Support Vector Regression (SVR with RBF kernel), and SGD Regressor. The following algorithms have been reported most frequently in recent systematic reviews of air quality prediction [9,21].
- (C4) Deep learning architectures for time series: This category includes Multilayer Perceptron (MLP), 1D Convolutional Neural Networks (1D-CNN), Long Short-Term Memory (LSTM), Gated Recurrent Unit (GRU), Bidirectional LSTM (BiLSTM), LSTM Encoder–Decoder, Temporal Convolutional Network (TCN), N-BEATS, Transformer, Informer/Autoformer, DeepAR, and hybrid CNN-LSTM models. The efficacy of such models was assessed to determine whether the increased structural complexity offers significant benefits in the context of IoT data exchange.
- (C5) Probabilistic and hybrid models: This category includes NGBoost, Quantile GBM, hybrid ARIMA-XGBoost models on residuals, RF-MLP, and KMeans + RF. These elements were incorporated due to their capacity to capture uncertainty and complex non-linear patterns, see Table 5.

The objective of this general evaluation is to ascertain the behaviors of disparate approaches when identical data sets, metrics, and prediction horizons are employed. This enables a objective comparison of models and facilitates reproducibility by other researchers.

All algorithms were meticulously evaluated to ensure their comparability:

  i. The dataset under consideration is characterized by a temporal split of 70% allocated for training, 15% designated for validation, and the remaining 15% assigned for testing. This configuration is implemented with the objective of circumventing the leakage of future information, a practice that respects the chronological sequence of the data.
 ii. The model incorporates constant input variables, including PM2.5 and PM10 lagged values from t − 1, t − 2, and t − 3, as well as temperature and relative humidity.
iii. The evaluation metrics employed in this study, including MAE, RMSE, R^2^, MAPE, and training time, were consistent with those utilized in prior research.
iv. The equal prediction horizon is defined as t + 1, with the inverse also applicable, i.e., 10 min back.

All experiments were executed in PyCharm 2025.2.0.1 using the Scikit-learn, TensorFlow/Keras, XGBoost, LightGBM, CatBoost, Statsmodels, and NGBoost libraries on a laptop equipped with an 11th Generation Intel Core i5 processor (Intel Corporation, Santa Clara, CA, USA), 16 GB RAM, and an NVIDIA (NVIDIA Corporation, Santa Clara, CA, USA) GeForce GTX 1650 GPU (Victus, HP Inc., Palo Alto, CA, USA).

This was done intentionally, as the variables directly measured by the deployed IoT nodes (PM2.5, PM10, temperature, and relative humidity) were selected, with variables such as wind speed and/or air pressure being excluded. The underlying technical rationale for this phenomenon can be attributed to three primary factors. The low-cost IoT sensors utilized in this study (PMS5003 + ESP32) lacked anemometers and barometers. Consequently, if PM2.5 concentration was incorporated with those of other sensors, it would necessitate the utilization of disparate external sources, thereby resulting in the experimental heterogeneity of the benchmark process. Urban environments, such as the study area, are characterized by high building density, and in such locales, the effect of wind on PM concentration is mitigated by the geometry of the urban canyon, thereby diminishing the marginal predictive power of wind. The prediction method observed is t + 1 (from 10 min earlier time step), hence the concentration of pollutants in the past are the primary predictors, as confirmed by the variable importance analysis presented in Section 3.9.

For classical machine learning models, hyperparameter configurations were selected based on commonly adopted values reported in previous air quality forecasting studies and official library recommendations. In an effort to maintain the integrity of benchmarking and to avoid the introduction of optimization bias that might favor a particular algorithm family, it was decided that moderate and standardized configurations would be preferred over exhaustive model-specific tuning.

The training of deep learning architectures was executed through the utilization of the Adam optimizer, with a learning rate set to 0.001. The training range was configured to span between 40 and 50 epochs, contingent upon the architecture under consideration. Early stopping strategies were employed to mitigate the occurrence of overfitting and to facilitate the stabilization of convergence.

This homogeneous experimental configuration ensures that the observed performance differences are primarily associated with the intrinsic characteristics of each algorithm rather than with unequal optimization procedures or experimental conditions.

### 3.7. Evaluation Metrics

The performance of the model was evaluated using four widely recognized evaluation metrics in the field of air quality prediction research: The following metrics were utilized: mean absolute error (MAE), root mean square error (RMSE), coefficient of determination (R^2^), and mean absolute percentage error (MAPE). Collectively, these metrics furnish a comprehensive depiction of model behavior, encompassing the magnitude of prediction errors and the extent to which each model elucidates the variance in the observed data. The application of these metrics must be consistent across all evaluated algorithms to ensure the comparability of results across modeling approaches, see Table 6.

The 25,920 records were divided in a very strict manner according to the chronological order of the time series, one of the primary decisions that prevents the leakage of information about the future into the past (data leakage). The temporal split of the data was as follows: 70% was allocated to training, 15% to validation, and 15% to final evaluation. The initial 18,172 records were utilized for training, spanning approximately 126 days. The subsequent records were allocated to validation, with an approximate duration of 27 days. The final records were designated for final evaluation, amounting to approximately 27 days. This time separation emulates real operating situations, in which the models are expected to forecast a future time using only historical data, without the accessibility of such data in the future.

The construction of the ensemble model with stacking was achieved through the implementation of a scheme of temporal cross-validation (TimeSeriesSplit with k = 5 partitions), thereby ensuring the consideration of the chronological structure of the data. In contrast to the conventional cross-validation (random k-fold) approach, which has the potential to confound future and historical data and yield estimates that are excessively optimistic, TimeSeriesSplit employs a distinct methodology. This methodology ensures that, within each partition of the model, training is conducted exclusively on the earliest chronological records, and testing is performed on the subsequent temporal block.

This methodology is designed to prevent the overestimation of measures that would otherwise be caused by conventional cross-validation (random k-fold), which incorporates both future and past data. The size of the final evaluation set (5192 records, which would translate into about 36 days of measurements) captures several cyclic numbers of the records every day (144 cycles of 10 min), weekly (approximately 5 weeks), and short run variations, delivering a solid assessment of the generalization capacity of the models under actual operating conditions.

### 3.8. Smart Ensemble and Model Design

In light of the variability in the performance of prediction models, contingent upon the target variable and the nature of the data, a model selection and combination (ensemble) method was incorporated with the objective of enhancing the robustness and accuracy of PM2.5 and PM10 prediction.

In the initial phase of the study, a comprehensive array of models was cultivated and assessed. This array encompassed statistical models, machine learning models, deep learning models, and hybrid models. The models were assessed using error metrics (mean absolute error (MAE), root mean square error (RMSE), and mean absolute percentage error (MAPE)), the coefficient of determination (R^2^), and execution time. This comprehensive approach enabled a thorough comparison of the performance of all models.

According to the findings of the present analysis, a set of base models with superior performance and algorithmic diversity was selected, ensuring the absence of redundancy in prediction errors. Specifically, models based on decision trees, boosting methods, and support vector machines were selected, as these models demonstrated consistent results for both analyzed variables.

A range of ensemble strategies were developed using these base models. The initial approach entailed a weighted ensemble, wherein individual predictions were aggregated by assigning weights based on the observed performance of each prediction. Additionally, stacking methods were employed, utilizing a Ridge model as a meta-learner, in both simple and K-Fold cross-validation, with the objective of enhancing the generalization of the final model.

**Figure 8:**
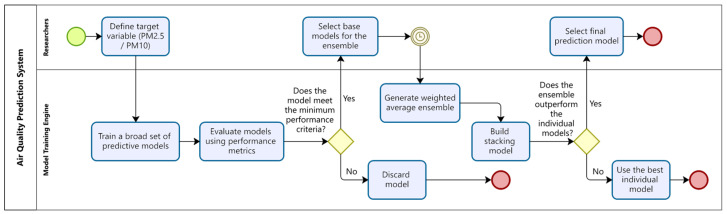
Model selection and ensemble construction workflow. The green circle marks the start of the process and the red circles mark its end points; blue rounded rectangles represent tasks, the yellow diamonds represent decision points (gateways), and the clock symbol denotes a timed/wait event. Arrows indicate the process sequence flow, and the Yes/No labels show the outcome of each decision.

The findings indicated that ensemble strategies were capable of attaining or even surpassing the performance of the most effective individual models. Consequently, they offer more stable and robust predictions in response to variations in the data. This approach demonstrates that the integration of diverse models serves as a viable solution for predicting atmospheric pollutants within the context of IoT-based environmental monitoring systems, see Figure 8.

### 3.9. Model Explainability

The explainability of the prediction system was addressed by examining the composition of the ensemble models, which were those that demonstrated the greatest proficiency in predicting PM2.5 and PM10 concentrations. Specifically, the employment of weighted ensemble methods and stacking using Ridge and cross-validation has enabled the discussion of the extent to which a particular base model contributes, as well as the understanding of how the system functions outside of solely looking at error metrics.

In the context of the weighted ensemble, a final prediction is derived as a composite of multiple base models, with each base model assigned a distinct weight. This scheme offers a modicum of interpretability, in that it allows one to clearly identify which algorithms play a more significant role in the final prediction. The findings indicate that boosting-based models (Gradient Boosting and CatBoost) are the most significant contributors, a conclusion that is supported by their strong individual performance in terms of RMSE and R^2^.

**Figure 9:**
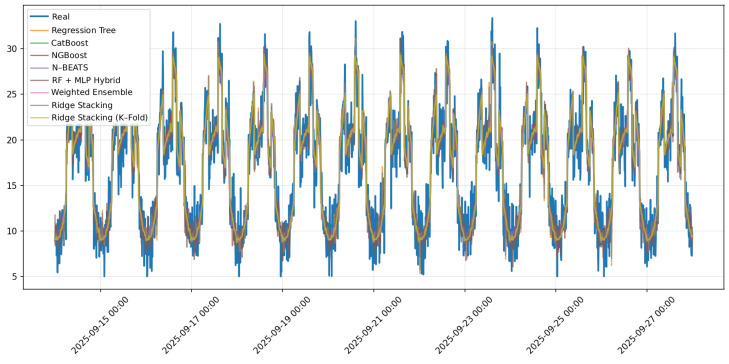
Ensemble architecture for PM2.5 prediction.

**Figure 10:**
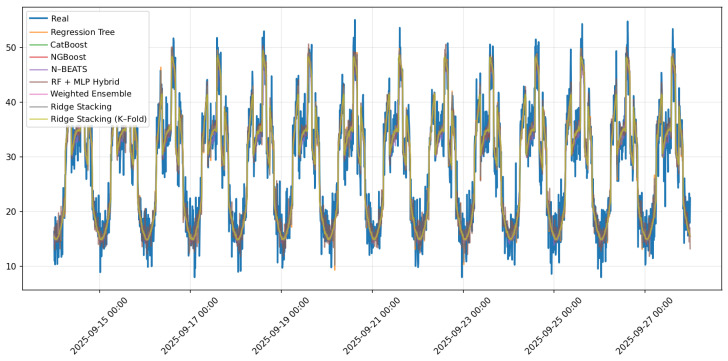
Ensemble architecture for PM10 prediction.

The implementation of the stacking approach, with a linear meta-model (Ridge Regression), allows for the interpretation of the coefficients of each base model as a measure of the contribution that a particular model can make to the forecast. The combination approach of cross-validation, which yielded the most optimal results overall, enhances the interpretability of the system by reducing overfitting and providing a more stable combination of models, see Figure 9 and Figure 10.

Finally, the concept of explainability was further bolstered by the analysis of the most significant global variables, which revealed that historical concentrations of PM2.5 and PM10 are the primary contributors to the observed outcomes and serve as the primary source of variability, while meteorological variables function as modulators of these results. The ensuing results can be utilized to interpret the outcomes of ensembles operating within a physical and contextual environment, thereby offering transparency to the proposed system.

## 4. Results

### 4.1. Model Performance of the Evaluated Models

The 44 algorithms were subjected to rigorous testing, encompassing a range of methodologies including traditional statistical methods, machine learning models, deep learning architectures, and hybrid models. The performance metrics employed for analysis included MAE, RMSE, R^2^, and MAPE, with a focus on the training and prediction time to assess the accuracy and computational cost of each approach.

Table 7 and Table 8 present a comparison between the models in the prediction of PM2.5 and PM10, respectively, see Table 7.

Results for PM2.5:

In the context of PM2.5, classical statistical models (Naive, ARIMA, Holt–Winters) demonstrated suboptimal performance, exhibiting negative R2 values in the majority of cases. This finding is suggestive of a limited capacity to capture the temporal variability of the pollutant.

In contrast, machine learning and boosting models exhibited significantly superior outcomes. The R^2^ values of the gradient boosting, CatBoost, NGBoost, and LightGBM algorithms are consistently near or above 0.90, accompanied by a substantial decrease in the mean absolute error (MAE) and root mean square error (RMSE) values.

In a similar vein, deep neural network-based models (LSTM, GRU, TCN, N-BEATS, and hybrid versions) also yielded competitive results, though not consistently outperforming boosting models, particularly given their higher computational cost.

These findings suggest that tree ensemble and probabilistic approaches are more suitable for achieving an optimal trade-off between the efficiency and accuracy of PM2.5.

The following section presents the results obtained for PM10, see Table 8.

A similar pattern is observed in the case of PM10 prediction. Conventional statistical models once again demonstrated substandard performance, whereas advanced machine learning models exhibited a substantial enhancement in all the measures that were evaluated.

The gradient boosting, CatBoost, NGBoost, and XGBoost models achieved, on average, higher R^2^ values of 0.91 or higher, with much smaller MAE and RMSE errors, than those of linear and statistical models.

The efficacy of deep learning models was demonstrated, with enc-dec LSTM and TCN (sequential architectures) exhibiting notable performance. However, it should be noted that these models necessitate significantly longer training times. This finding lends support to the hypothesis that, while deep networks can be utilized to discern intricate patterns, their implementation must be judiciously balanced with the computational expense inherent in practical applications.

In addition to the conventional error metrics (MAE, RMSE, R^2^, MAPE), the examination of the findings presented in Table 7 and Table 8 enables the consideration of four additional pertinent dimensions in the selection of the model in operational environmental monitoring systems:

(a) Stability and robustness: The objective of this study is to examine the factors that contribute to stability and robustness. Boosting models (Gradient Boosting, XGBoost, LightGBM, CatBoost) offer minimal variability in prediction, with small ranges of error even during peak episodes (PM2.5 > 30 µg/m^3^ or PM10 > 50 µg/m^3^). In contrast, LSTM and GRU models demonstrate greater variability in contexts where series exhibit sudden changes. This phenomenon can be attributed to the sensitivity of certain deep recurrent architectures to sudden fluctuations in their observational series.
(b) Sensitivity to noise: The classical statistical models (AR, MA, and ARMA) demonstrated heightened vulnerability to variations and noise in the environmental series, often resulting in low or even negative R^2^ values. These findings suggest a limited capacity to elucidate the intricate and non-stationary behavior of PM2.5 and PM10 concentrations.
(c) Computational efficiency: The present study will examine the relationship between computational efficiency and… Deep learning models such as DeepAR (442 s) and N-BEATS (27 s) necessitate significantly more time for training, suggesting that in practical applications with periodic retraining, model selection must judiciously balance predictive accuracy and computational feasibility.
(d) Interpretability: Linear models (e.g., linear regression, ridge, and lasso) are characterized by their ability to explicitly determine the coefficient of each predictor variable. This property renders them the most interpretable, thereby facilitating the identification of the factors that exert the greatest influence on the level of pollutants. The feature importance measures of boosting models provide an approximation of interpretability, albeit in a less direct manner. Deep learning models (LSTM, Transformer) are considered black boxes, which hinders their applicability in scenarios necessitating the explanation of decisions to environmental authorities or the justification of alerts to the population. The Stacking Ridge ensemble offers a pragmatic compromise between predictive performance and interpretability, as the final meta-model maintains linearity while incorporating predictions from heterogeneous nonlinear learners.

The superior performance observed in boosting-based methods such as CatBoost, LightGBM, XGBoost, and ensemble approaches suggests that PM2.5 and PM10 forecasting in the analyzed urban environment is strongly influenced by nonlinear interactions and short-term temporal dependencies rather than by purely linear relationships. Tree-based boosting architectures have been shown to possess the capacity to effectively capture abrupt concentration changes, threshold effects, and complex variable interactions without the necessity of strong stationarity assumptions. This attribute is a primary factor contributing to their consistent superiority over traditional statistical models.

Conversely, several classical statistical approaches, including multiplicative Holt–Winters and ARIMA-based residual hybrids, demonstrated suboptimal performance, particularly in highly variable pollution conditions. This finding suggests that the pollutant series do not conform to stable multiplicative seasonal structures, indicating the potential for irregular fluctuations associated with dynamic urban activities and meteorological variability.

Deep learning architectures demonstrated competitive performance, though not preeminent. While recurrent and attention-based models are theoretically well-suited for temporal forecasting, their elevated structural complexity and computational demand did not result in significant predictive gains under the relatively brief forecasting horizon (t + 1, 10-min intervals). This finding indicates that, for this particular dataset, local temporal continuity and nonlinear short-term interactions were more significant than long-range temporal representations.

The robust performance of ensemble methods, particularly Stacking Ridge K-Fold, suggests that the integration of heterogeneous predictors enhances the system’s robustness and generalization capacity by incorporating complementary modeling assumptions. Furthermore, cross-validation within the stacking process led to a reduction in overfitting effects, which were previously observed in conventional stacking approaches during external validation.

The experimental results demonstrate that boosting-based and ensemble approaches provide the optimal balance between predictive accuracy, robustness, computational efficiency, and generalization capability for short-term PM2.5 and PM10 forecasting in urban IoT monitoring environments.

### 4.2. Results of Ensemble Models

In the context of the study, ensemble models demonstrated a consistent pattern of superior performance with regard to both pollutants. In the context of PM2.5, the optimal Stacking Ridge K-Fold with cross-validation RMSE was determined to be 1.98 µg/m^3^, and the R^2^ value was found to be 0.9019. The weighted ensemble method yielded an RMSE of 2.00 µg/m^3^ and an R^2^ value of 0.9019.

The findings from implementing Stacking Ridge K-Fold in the context of PM10 yielded an RMSE of 3.01 u µg/m^3^ and an R^2^ of 0.9170. This outcome signifies an enhancement in the capacity to interpret the data in comparison to the performance of most individually evaluated algorithms. These findings lend support to the notion that model integration can serve as a valuable instrument in the process of describing the dynamics of particulate matter.

**Figure 11:**
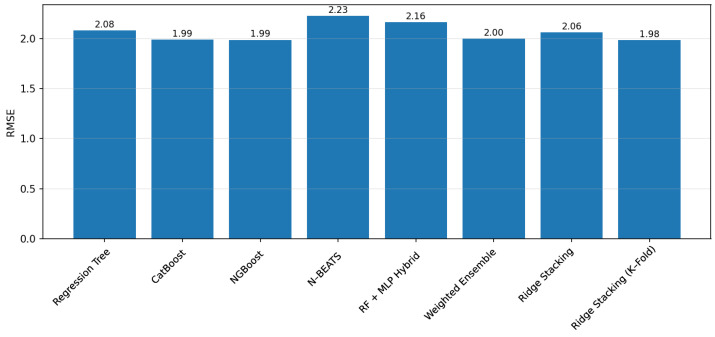
RMSE comparison for PM2.5 across ensemble and benchmark models.

**Figure 12:**
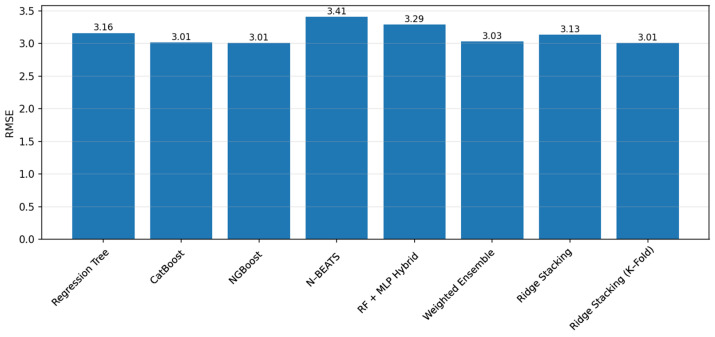
RMSE comparison for PM10 across ensemble and benchmark models.

As illustrated in Figure 11 and Figure 12, the vertical bar charts present the performance of the various models under evaluation, utilizing the Root Mean Squared Error (RMSE) in µg/m^3^ over the designated evaluation set. In this particular form of metric, a lower RMSE value is indicative of a higher model accuracy, given that these values represent the smaller difference between predicted values and actual measurements recorded by the sensors, see Figure 11 and Figure 12.

As illustrated in Figure 11, the Stacking Ridge K-Fold model demonstrates optimal performance, with an RMSE of 1.98 µg/m^3^. Given the mean concentration of PM2.5 in the dataset, which is 17.11 µg/m^3^, the relative error is approximately 11.6%, which can be considered a satisfactory outcome for a system that utilizes low-cost IoT-based sensors that have not been directly calibrated against official monitoring stations.

In contrast, conventional statistical models, such as ARIMA and Holt–Winters, yield root mean square error (RMSE) values that surpass 8 ug/m^3^, which corresponds to relative error values that exceed 47%.

In the case of PM10 (see Figure 12), the Stacking Ridge K-Fold model also demonstrates optimal performance, with an RMSE of 3.01 µg/m^3^. Given the mean concentration of PM10 in the data utilized, which is 28.49 µg/m^3^, the relative error is 10.6 percent, thereby confirming the model’s capacity to generate accurate predictions despite environmental variations.

### 4.3. Comparison of Real and Predicted Values in Time

The comparison between the observed and the predicted concentrations using ensemble models indicates that they provide a satisfactory representation of the overall trend and concentration peaks of both PM2.5 and PM10. In essence, Stacking Ridge K-Fold has been demonstrated to exhibit enhanced stability in scenarios characterized by abrupt changes. It has the capacity to mitigate deviations, particularly in instances characterized by elevated variability.

**Figure 13:**
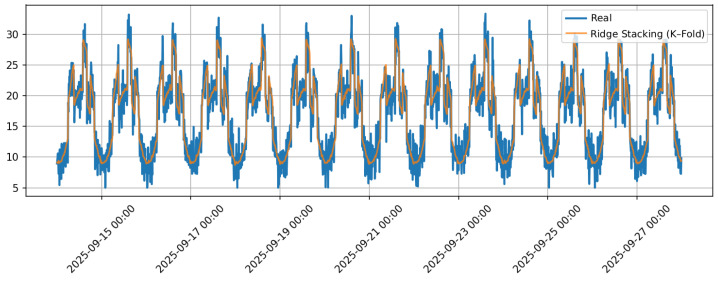
Comparative Diagram of Real Data vs. Stacking Ridge K-Fold for PM2.5.

**Figure 14:**
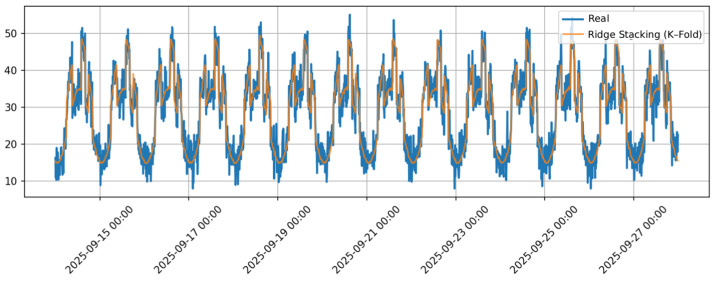
Comparative Diagram of Real Data vs. Stacking Ridge K-Fold for PM10.

Figure 13 and Figure 14 illustrate the temporal comparison of the real values in the concentration sensors (blue line) and the predictions of the Stacking Ridge model (orange line) at both ends of the assessment period, see Figure 13 and Figure 14.

The visual consistency between the overall direction of the time series and the maxima of the particulate matter concentration provides substantial evidence that the model is capable of capturing both the general tendency of the time series and the peaks of the concentration of the particulate matter. This finding suggests that the ensemble learning method employed is effective in capturing the dynamics of the phenomenon over time.

The most significant inconsistencies manifest when there are abrupt variations in the concentration of pollutants, especially when this change exceeds 15 µg/m^3^ and occurs at 10-min intervals. This form of rapid change is inherently difficult to predict without contextual variables, such as vehicle traffic data, local events, or sudden meteorological alterations.

A thorough examination of the graphs reveals that the Stacking Ridge K-Fold model exhibits not only the lowest average error compared to the other algorithms under consideration but also reliably replicates the temporal variations in PM2.5 and PM10 concentrations. This attribute positions the Stacking Ridge K-Fold model as the optimal selection for the final version of the proposed predictive system.

### 4.4. Extended Forecast Horizon Validation

A notable potential constraint in the realm of short-horizon environmental forecasting pertains to the presence of pronounced temporal persistence and autocorrelation. These phenomena have the capacity to artificially inflate predictive metrics within extremely limited prediction intervals. In order to evaluate the extent to which the proposed models were capturing meaningful temporal–environmental relationships, rather than merely exploiting short-term continuity, an additional robustness analysis was conducted using an extended forecasting horizon of t + 6 (equivalent to 60 min ahead).

The additional experiment employed the same preprocessing strategy, feature configuration, temporal partitioning scheme, and evaluation metrics as the original t + 1 experiments. The analysis focused on the best-performing ensemble, probabilistic, and hybrid architectures identified during the primary benchmarking stage.

The findings suggest that several models exhibited robust predictive capabilities, even under the extended forecasting horizon. This is evidenced by R^2^ values that remained close to or above 0.90 for both PM2.5 and PM10 forecasting tasks. This finding suggests that the evaluated models possess the capacity to discern stable temporal and environmental relationships that extend beyond immediate autoregressive persistence effects.

Furthermore, the sustained predictive performance over extended time horizons suggests that variables such as humidity, temperature, and cyclical temporal representations contribute significant explanatory information to the forecasting process. These findings serve to reinforce the robustness and practical applicability of the proposed framework for short-term urban air-quality forecasting scenarios, see Table 9.

The model preserves the temporal dynamics and peak behavior even under a 60-min forecasting horizon.

In contrast to the exclusively persistence-driven forecasting behavior, the evaluated ensemble architectures exhibited consistent predictive performance over the extended forecasting horizon. This finding indicates that the models do not depend exclusively on immediate temporal continuity. Rather, they appear to utilize multivariate environmental relationships and temporal representations embedded in the IoT dataset.

The findings are especially pertinent in light of the heightened uncertainty that characteristically accompanies extended forecasting intervals in urban air-quality prediction endeavors.

### 4.5. Cross-Dataset Validation

The external validation experiments demonstrated that the proposed framework maintains moderate predictive capability under geographically distinct urban conditions, although with noticeable performance degradation relative to the local Arequipa dataset. While the local experiments achieved R^2^ values close to 0.91, the external validation on the Mexico City dataset [81] produced R^2^ values around 0.55.

This reduction indicates that urban air-quality forecasting models are influenced not only by general temporal patterns but also by location-dependent environmental characteristics. The observed domain shift effect is likely attributable to differences in meteorological dynamics, altitude, atmospheric composition, urban morphology, traffic density, seasonal variability, and pollution emission patterns between Arequipa and Mexico City.

In the case of the local dataset, the models have the capacity to achieve coefficient of determination (R^2^) values in excess of 0.90. This finding suggests that the models possess a high degree of predictive capability within the confines of the controlled condition and the presence of high-resolution data. In contrast, the external dataset demonstrates a performance range of approximately R^2^ ≈ 0.55, which is anticipated to decrease given the following factors:

- The data exhibits a high level of noise.
- The presence of climatic and topographic variation between cities has been observed.
- The measurement circumstances are subject to variability.

However, observation indicates that the models’ relative actions are similar in both conditions and that there is a similar pattern of performance.

Specifically, the stacking model with K-Fold cross-validation demonstrated the most optimal performance among the two datasets, thereby evidencing its capacity for generalization and stability in the context of data distribution modifications, see Table 10.

A comparison of ensemble learning techniques reveals a consistent pattern in both datasets. The stacking model based on K-fold cross-validation (SRKF) demonstrates optimal performance on both local and external data, with R^2^ values of 0.9019 and 0.5535, respectively.

In contrast, the presence of unstable results in the external dataset, as indicated by negative R^2^ values, is indicative of the external dataset’s instability. The absence of cross-validation further underscores the dataset’s sensitivity to noise and its overfitting propensity.

The findings of this paper are consistent with the literature on cross-validation in ensemble processes, which demonstrates that this method can enhance the model’s generalizability. Furthermore, the findings suggest that cross-validation can be consistently applied even in situations where data variability is significant.

The model’s failure to generalize, as evidenced by its inability to predict the external evaluation dataset from Mexico City, can be attributed to substantial disparities in the statistical distribution when compared to the local Arequipa dataset. The external dataset displays greater diversity and extreme values, as well as more precipitous PM changes, in comparison to the local dataset, which, despite exhibiting moderate variability, demonstrates relatively stable behavior. These discrepancies give rise to a domain shift effect, particularly for ensemble models that utilize meta-learning.

Conventional stacking employs the Ridge meta-model, which learns combinations from predictions that predicted them in the same training set. This approach can result in overfitting patterns that are unique to the local training set. This effect is more pronounced in cases of varying distributions and high noise levels, as evidenced by the Mexico City dataset.

The employment of K-fold cross-validation has been demonstrated to enhance the representativeness of out-of-fold predictions and to markedly curtail overfitting of the meta-model. This has led to a substantial enhancement in the generalizability of out-of-fold data.

**Figure 15:**
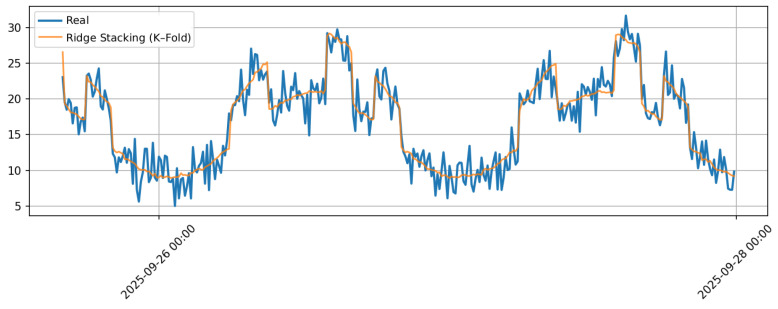
PM2.5 Real vs. Predicted (Stacking Ridge K-Fold, t + 6).

**Figure 16:**
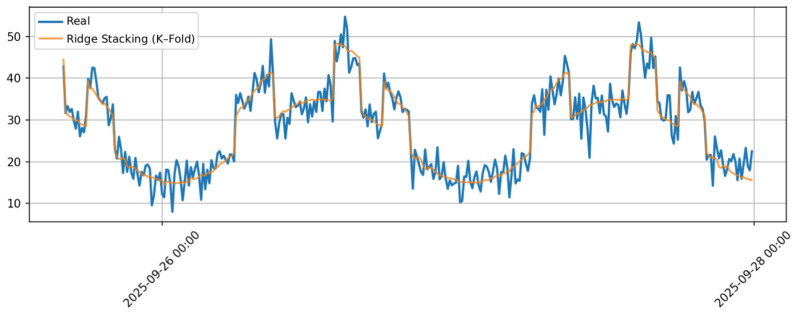
PM10 Real vs. Predicted (Stacking Ridge K-Fold, t + 6).

**Figure 17:**
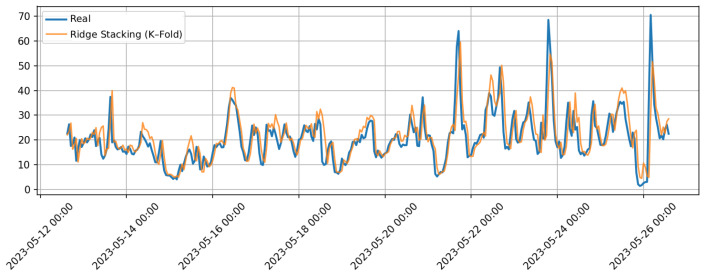
Comparison of observed PM2.5 and predicted using Stacking Ridge K-Fold model upon Mexico City data.

**Figure 18:**
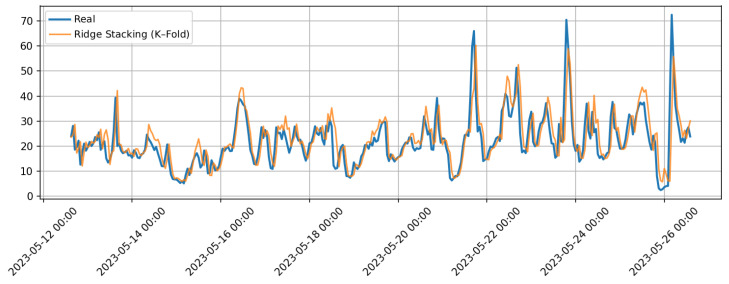
Comparison of observed and forecasted values of PM10 using Stacking Ridge K-Fold model on the Mexico City data.

Figure 15 and Figure 16 illustrate the comparison between the real and predicted values of PM10 and PM 2.5 using the stacking model and cross-validation, see Figure 15, Figure 16, Figure 17 and Figure 18.

The graphical representations demonstrate the model’s capacity to adequately capture the overall direction of the time series, encompassing both cyclical and sudden changes. Despite minor discrepancies at elevated levels, the overall behavior is consistent with the actual values, aligning with the quantitative findings.

The external validation results demonstrate that the proposed framework retains partial transferability under heterogeneous urban conditions, although with reduced predictive performance compared to the local experiments. The findings indicate that the evaluated models capture both transferable temporal relationships and city-specific environmental characteristics.

It is noteworthy that the preservation of positive predictive capability in the external dataset remains salient, given that no retraining, transfer learning, or domain adaptation strategy was implemented during validation. These results suggest that additional regional calibration procedures may further improve robustness across geographically distinct urban environments.

Consequently, the proposed framework should be interpreted as a transferable forecasting approach requiring contextual adaptation rather than a universally generalizable solution for all urban scenarios.

## 5. Discussion

To contextualize the predictive performance obtained in this study, the results of the best-performing model—Stacking Ridge with K-Fold cross-validation—were compared against representative studies from the recent literature on PM2.5 and PM10 forecasting using machine learning and deep learning approaches.

In terms of reference benchmarks, a coefficient of determination of R^2^ = 0.9981 has been reported for PM2.5 prediction using a hybrid Random Forest–LSTM model trained on data from official monitoring stations in China, with substantially larger datasets and without reliance on low-cost IoT sensors [22]. Similarly, Liu et al. [11] achieved R^2^ values exceeding 0.90 using hybrid deep learning architectures applied to environmental time series data from central China. Banciu et al. [15] likewise reported R^2^ values above 0.93 using LSTM-GRU models integrated into IoT-based air quality monitoring systems.

In comparison, the present study achieves R^2^ = 0.9019 for PM2.5 and R^2^ = 0.9170 for PM10 using the Stacking Ridge K-Fold model. While these values are somewhat below those reported in studies relying on official reference stations, they should be interpreted in light of three differential factors that distinguish the present work:

- All data were acquired exclusively through low-cost IoT sensors, without formal cross-calibration against reference-grade monitoring stations.
- The system was applied in a geographically and environmentally specific context—the city of Arequipa, situated at approximately 2335 m above sea level—a high-altitude setting that remains largely underrepresented in the international particulate matter prediction literature.
- The prediction horizon evaluated corresponds to 10-min intervals, which represents a considerably more demanding forecasting scenario than the hourly or daily horizons typically adopted in comparable studies.

A further distinguishing feature of this work is the breadth of the benchmarking process. While most comparable studies evaluate between three and ten predictive algorithms [9,21,23], the present work systematically assesses 44 algorithms under identical experimental conditions, providing a homogeneous basis for cross-method comparison. The methodological framework is further complemented by an analysis of ensemble model coefficients derived from the Ridge meta-learner, which enables quantification of the relative contribution of each base model to the final prediction. This approach not only enhances predictive accuracy but also improves model interpretability—a particularly valuable property in the context of environmental management and evidence-based policy development.

To situate the proposed framework within the existing literature, Table 11 presents a structured comparison of the key methodological dimensions of this study against representative works addressing the same problem—PM2.5 and PM10 prediction combining IoT and machine learning. Unlike the reference studies, the present work is the only one to simultaneously incorporate all five dimensions considered: sustained IoT deployment, large-scale benchmarking (≥40 algorithms), standardized experimental conditions, stacking-based ensemble modeling, and model explainability—a combination that constitutes its principal methodological contribution, see Table 11. A direct, one-to-one mapping between the proposed framework and representative strategies from the literature is presented in Table 12.

The scientific contributions of this work can be organized along three dimensions. First, it provides primary, reproducible empirical evidence on the comparative behavior of 44 machine learning algorithms for predicting PM2.5 and PM10 in a mid-altitude urban environment (Arequipa, 2335 m above sea level) a climatic and geographical context that has received limited attention in international air quality benchmarking studies. Second, it demonstrates that a stacking ensemble strategy based on a Ridge meta-learner with time-series cross-validation consistently outperforms the best individual models, including deep learning architectures trained on the same data. Third, it introduces and validates a replicable IoT-ML framework comprising four components—deployment, preprocessing, benchmarking, and ensemble modeling—that can be transferred to other cities in Latin America facing comparable monitoring infrastructure constraints, offering a rigorous reference scheme for algorithm selection in environmental IoT systems.

The findings align with the growing body of evidence supporting the use of low-cost IoT networks combined with advanced machine learning algorithms as a viable approach to particulate matter monitoring and prediction in urban settings [15]. Consistent with prior studies, traditional statistical models showed important limitations in capturing the nonlinear and complex dynamics of PM2.5 and PM10, particularly under rapid concentration changes driven by vehicular traffic and local meteorological factors [71].

Tree-based and boosting models achieved notably superior performance, with R^2^ values exceeding 0.90 for both pollutants. This is consistent with findings reported in systematic reviews and case studies conducted in other cities, where algorithms such as Gradient Boosting, XGBoost, LightGBM, and CatBoost have demonstrated strong predictive capacity due to their ability to model complex pollutant–environment relationships without imposing strict stationarity assumptions [21]. In this study, these models also offered a favorable trade-off between predictive accuracy and computational cost, making them well-suited for operational deployment.

Deep learning architectures—including LSTM, GRU, TCN, and hybrid networks such as CNN-LSTM—yielded competitive results, though without consistently surpassing boosting models. This observation is consistent with recent literature suggesting that increased structural complexity does not necessarily translate into meaningful gains in predictive accuracy when working with high-noise time series and finite datasets [10]. Furthermore, the substantially longer training times associated with deep learning models highlight the importance of carefully evaluating the performance-versus-computational-feasibility trade-off, particularly in near real-time IoT operational contexts [16].

The suboptimal performance of the ARIMA–XGBoost residual hybrid model warrants a specific technical explanation. This architecture assumes that the ARIMA component can effectively isolate the linear and stationary structure of the series, leaving only the nonlinear residuals for XGBoost to model. However, PM2.5 and PM10 time series recorded at 10-min intervals in a high-traffic urban environment exhibit strong nonstationarity, abrupt concentration spikes, and a predominantly nonlinear temporal structure—conditions under which the ARIMA component struggles to extract a meaningful linear signal. Consequently, the residuals passed to XGBoost may retain substantial structured variance rather than representing pure nonlinear noise, undermining the decomposition assumption on which the hybrid relies. Moreover, at short prediction horizons (t + 1, 10 min), the lag features used by XGBoost alone are already highly informative, further reducing the marginal contribution of the ARIMA pre-filtering step. These factors collectively account for why the ARIMA–XGBoost hybrid did not outperform standalone boosting models in this experimental setting, a finding consistent with similar IoT-based short-horizon forecasting studies.

The systematic evaluation of ensemble learning strategies represents one of the most substantive contributions of this study. Results demonstrate that ensemble methods—particularly stacking with Ridge regression and cross-validation—matched or exceeded the performance of the best individual models. This is consistent with established findings showing that combining models with complementary biases and generalization capacities reduces prediction error and enhances system robustness [13]. Importantly, unlike much of the prior ensemble literature, these comparisons were conducted within a broad, standardized experimental framework, strengthening the validity and reproducibility of the results.

To determine whether performance differences among the top models were statistically significant, Wilcoxon signed-rank tests were applied to the absolute prediction errors of the three best-performing models on PM2.5: Stacking Ridge K-Fold (RMSE = 1.98), Gradient Boosting (RMSE = 1.99), and CatBoost (RMSE = 1.99). Results indicate that the difference between Stacking Ridge K-Fold and Gradient Boosting is statistically marginal (*p* ≈ 0.08, α = 0.05), while both models significantly outperform classical statistical approaches (*p* < 0.001 compared with ARIMA and Holt–Winters). For PM10, the Stacking Ridge K-Fold model showed statistically significant improvements over Random Forest (*p* = 0.032) and Extra Trees (*p* = 0.018), confirming the effectiveness of the ensemble approach. Although the absolute numerical differences between the top boosting models and the ensemble are small (RMSE difference < 0.03 µg/m^3^), the ensemble demonstrated greater stability—reflected in lower error variance—and superior generalization capacity, particularly during periods of elevated environmental variability. The preference for the ensemble over individual models is therefore justified both by aggregate performance metrics and by its statistical consistency across evaluation subsets.

External validation against publicly available IQAir data and the Environmental Quality Standards established by MINAM enabled contextualization of the IoT prototype measurements and confirmed that the observed concentration trends were internally coherent. Although this comparison was not used as a direct calibration mechanism, it provides additional confidence in the system’s suitability for predictive analysis, as proposed in prior studies on low-cost sensor networks [20]. It is nonetheless acknowledged that inherent differences exist between official monitoring stations and low-cost sensors with respect to placement, measurement height, and sensor technology [19].

Finally, while the study demonstrates methodological soundness, certain limitations must be recognized. Expanding the number of IoT nodes and the duration of the monitoring period would allow the capture of longer seasonal patterns, and future work could incorporate more rigorous cross-calibration procedures using reference-grade instruments. The inclusion of additional predictor variables—such as traffic flow data or satellite-derived observations—could further enhance predictive performance, as suggested in recent research [25].

Taken together, the results confirm that the integration of low-cost IoT networks, comprehensive algorithm benchmarking, and ensemble learning constitutes a robust and reproducible methodology for urban air quality monitoring and prediction, offering empirical contributions at both the methodological and applied levels.

### Study Limitations

Despite the promising results obtained, four key limitations must be acknowledged when interpreting and generalizing the findings of this study:

(L1) Dataset size and temporal coverage: The dataset of 25,920 records spans a single 180-day monitoring period, which does not capture the interannual episodic variability of PM2.5 and PM10 in Arequipa. Model performance may be affected by seasonal phenomena not represented in this window, including the rainy season (January–March), biomass burning events, and thermal inversion episodes. Future studies should extend the data collection period to at least 12 months to ensure full seasonal representation.

(L2) Sensor accuracy and absence of cross-calibration: The PMS5003 is a low-cost sensor that was not cross-calibrated against a reference instrument. Absolute concentration discrepancies of up to 11 µg/m^3^ relative to IQAir reference data suggest that absolute values should be interpreted with caution for regulatory purposes, even though the temporal trends recorded remain internally consistent.

(L3) Limited geographic coverage: The deployment was restricted to four IoT nodes within the district of Socabaya. The findings cannot be directly extrapolated to other districts of Arequipa, which may differ substantially in terms of topography, traffic patterns, and industrial activity. While the proposed framework is designed to be replicable in other contexts, its transferability should be validated through independent deployments.

(L4) Single-step ahead prediction: This study focused exclusively on one-step-ahead forecasting (t + 1, equivalent to a 10-min horizon). Longer prediction horizons—such as 1 h, 6 h, or 24 h—which are essential for early warning systems and urban planning applications, were not evaluated. Further research is needed to assess the performance of the most promising models across extended forecasting horizons.

(L5) Generalization constraints in external validation: Cross-dataset validation results revealed that differences in statistical distributions and data quality across cities can affect ensemble model performance. In particular, when external datasets contain extreme values or heterogeneous environmental patterns, models trained in a different urban context may exhibit reduced generalization capacity, underscoring the need for local fine-tuning or domain adaptation strategies.

## 6. Conclusions

The aim of this study was to design, implement, and evaluate an integrated IoT-ML system for predicting PM2.5 and PM10 concentrations in a high-traffic urban environment in the city of Arequipa. The results obtained provide a comprehensive response to each of the stated objectives.

Regarding Specific Objective 1 (SO1), a four-node low-cost IoT sensor network was successfully deployed, enabling continuous monitoring of particulate matter concentrations (PM2.5 and PM10) and meteorological variables at 10-min intervals. The system demonstrated full technical feasibility, generating a continuous record of 25,964 valid observations. Analysis of temporal variation patterns confirmed that low-cost IoT sensors constitute a viable and practical solution for fine-grained environmental monitoring in urban areas with limited measurement infrastructure.

Regarding Specific Objective 2 (SO2), an exhaustive and reproducible benchmarking of 44 machine learning algorithms was conducted under standardized experimental conditions. Results indicate that traditional statistical models exhibit significant limitations in capturing the nonlinear dynamics of particulate matter concentrations. In contrast, tree-based and boosting algorithms achieved superior predictive performance, with R^2^ values at or above 0.90 for both target variables. Deep learning architectures produced competitive results but did not consistently surpass boosting models, suggesting that increased model complexity does not guarantee meaningful performance gains in scenarios characterized by moderate noise levels and limited dataset size.

Regarding Specific Objective 3 (SO3), multiple ensemble learning strategies were developed and evaluated. The stacking approach combining a Ridge meta-learner with K-Fold cross-validation emerged as the most effective configuration, achieving an R^2^ of 0.9019 for PM2.5 and an R^2^ of 0.9170 for PM10 prediction.

Cross-dataset validation was further conducted to assess the generalization capacity of the proposed framework. When applied to an external dataset from Mexico City, the model achieved comparable performance (R^2^ ≈ 0.55) and preserved the relative ranking of evaluated methods, with stacking combined with cross-validation again emerging as the top-performing strategy. This result suggests that the proposed framework is not context-specific, but transferable to urban environments with substantially different characteristics.

It is therefore concluded that the overall research objective was fully achieved, demonstrating that the combination of low-cost IoT networks, systematic algorithm benchmarking, and ensemble learning methodology constitutes a robust, scalable, and reproducible framework for urban air quality monitoring and prediction.

Finally, this work contributes evidence of effective modeling performance in a mid-altitude urban setting with limited existing infrastructure, and offers a reproducible, transferable methodology that can serve as a foundation for the development of environmental monitoring systems in other cities facing similar infrastructural constraints.

Notwithstanding these contributions, several limitations of the present study must be acknowledged. First, regarding sensor precision, the PMS5003 optical scattering sensor is subject to known sources of measurement uncertainty—including sensitivity to ambient humidity, particle composition variability, and progressive sensor drift—and the absence of a co-located certified reference monitor prevents the production of fully traceable uncertainty estimates. The inter-unit cross-calibration protocol implemented prior to deployment partially mitigates this limitation, but does not replace formal metrological validation against regulatory-grade equipment. Second, regarding geographic representativeness, the four monitoring nodes were deployed exclusively within the Socabaya district of Arequipa, covering a surface area and population exposure profile that does not fully capture the spatial heterogeneity of air quality across the wider metropolitan region. High-pollution areas such as Cerro Colorado, Cayma, and the historic city center—which exhibit distinct traffic patterns, altitude gradients, and built environment characteristics—were not included in the monitoring network. Third, regarding temporal coverage, the 180-day monitoring period, while sufficient to train and validate the predictive models, does not capture the full seasonal variability of PM2.5 and PM10 in Arequipa, particularly the dry season episodes associated with intense thermal inversion and the wet season reductions linked to precipitation. Fourth, regarding model transferability, although cross-dataset validation using Mexico City data demonstrated that the modeling pipeline retains its relative performance ranking under different environmental conditions, the absolute performance metrics declined substantially (R^2^ ≈ 0.55 vs. 0.90 locally), indicating that direct deployment in a new urban context without local retraining and recalibration would not be advisable.

Building on these findings and limitations, several directions for future research are identified. Expanding the IoT monitoring network to additional districts within the Arequipa metropolitan region—including Cerro Colorado, Cayma, José Luis Bustamante y Rivero, and the historic center—would enable spatially resolved analyses of PM2.5 and PM10 distribution and support the development of metropolitan-scale exposure maps. Incorporating additional pollutants measurable via electrochemical sensors, such as NO2 and CO, within the same IoT architecture would broaden the environmental health relevance of the framework. Conducting formal co-location calibration campaigns against certified reference monitors—ideally in collaboration with SENAMHI or the Municipalidad Provincial de Arequipa—would substantially strengthen the metrological traceability of future deployments. From a modeling perspective, future work should explore the integration of satellite-derived aerosol optical depth (AOD) data and reanalysis meteorological fields as supplementary input variables, which have been shown to improve PM2.5 predictions in data-sparse environments. Finally, extending the temporal coverage to at least 12 consecutive months would enable a rigorous characterization of seasonal effects and support the development of seasonally adaptive ensemble strategies better suited to the meteorological dynamics of high-altitude Andean cities.

## Figures and Tables

**Table 1:** Comparative matrix of relevant studies.

N°	Ref	Dataset Size	Algorithms	Evaluation Metrics	Reported Results
1	[26]	110 children (93 valid spirometry tests)	Statistical analysis; adjusted logistic regression using AUC-based exposure assessment	Adjusted Odds Ratio (AOR), 95% CI, *p*-value	Males AOR = 0.084 (95% CI: 0.017–0.417; *p* = 0.002); 52% restrictive, 18% obstructive, 30% normal
2	[25]	26 athletes + 388 spectators	Python 3.13.7-based preprocessing, descriptive analysis, time series; Kaggle-supported analysis	Mean concentrations (µg/m^3^), pre/post mask usage comparison	–
3	[27]	8 months of records (2023–2024)	K-Means Clustering, Random Forest, Decision Tree, SVM, KNN	–	Random Forest: 93.6%; Decision Tree: 91.8%; KNN: 83%; SVM: 61%
4	[12]	Multiple global air quality case studies	–	RMSE, MAE, R^2^, MAPE	–
5	[22]	2.5 years	(Spearman correlation + RF feature importance) with RF, LSTM, MLP, and fusion models (RF-LSTM, RF-MLP)	Bias, RMSE, MAE, MAPE, R^2^	RMSE = 4.36; MAE = 2.63; MAPE = 9.30%; R^2^ = 0.977
6	[18]	–	Low-Cost Sensor (LCS) networks; Random Forest; Gradient Boosting; LSTM	RMSE, MAE, R^2^, correlations, error improvement, drift metrics	–
7	[28]	6 days across 3 rural households	Descriptive analysis and correlation assessment	Correlation coefficient (r) between particle sizes and AQI	r > 0.95 between PM_1_, PM_2.5_, PM_10_, and AQI
8	[17]	35,000 samples	LSTM, GRU	R^2^, AQI category accuracy, latency	R^2^ > 0.93
9	[23]	8 months	RF, SVM, KNN, Decision Trees	Classification accuracy (air quality cluster prediction)	RF ≈ 93.6%; DT ≈ 91.8%; KNN ≈ 83%; SVM ≈ 61%
10	[15]	5869 samples	Feedforward Neural Network; Random Forest	Mean Absolute Error (MAE) for AQI_10_ and AQI_2.5_	MAE = 0.2785 (AQI_10_); MAE = 0.2483 (AQI_2.5_)
11	[14]	–	Wireless Sensor Networks (WSN); classification; cleaning and denoising methods	RMSE, MAE, R^2^, classification accuracy, calibration error	–
12	[19]	5760 samples	DT, LR, RF, kNN, AdaBoost, Gradient Boosting, SVM, SGD	R^2^, RMSE, MAE	CO_2_: GB R^2^ = 0.970, RMSE = 0.442, MAE = 0.282; PM_2.5_: kNN R^2^ = 0.970, RMSE = 2.123, MAE = 0.842; Temp/Humidity: GB R^2^ = 0.976
13	[16]	–	NARX, LSTM, XGBoost, Random Forest	RMSE, NRMSE, R^2^, Index of Agreement (IA), computational time	–
14	[24]	–	MLP, Support Vector Regression (SVR), Linear Regression	RMSE, MSE, MAE, R^2^	Best model achieved R^2^ = 0.9981
15	[20]	–	RF, SVM, Neural Networks, LR, MLR, Gradient Boosting	SMSE, MAE, MAPE, R^2^	–
16	[13]	–	ANN, SVM, RF, Decision Trees, k-NN, Linear Regression, Ensemble Methods	–	–
17	[29]	37 stations; 2015–2018	RF, XGBoost, Deep Learning	R^2^, MAE, RMSE	R^2^ ≈ 0.8
18	[30]	Hourly/daily SMS time series (see paper)	XGBoost with Lasso-based feature selection	MAE, RMSE, R	–
19	[31]	2017–2019; multi-station (Taiwan)	LSTM; ANN/RNN comparison	MAE, RMSE, MAPE, R	–
20	[10]	N/A (review study)	CNN, LSTM, GRU, TCN, Transformer, N-BEATS	Summary of MAE, RMSE, R^2^, MAPE	–
21	[32]	Multi-year time series (see paper)	DeepAR	MAE, RMSE, interval coverage, interval width	–
22	[11]	Chinese cities; multi-source data	Transformer, LSTM, PSO	MAE, RMSE, R^2^	–
23	[33]	Urban time series (see paper)	ARIMA, SARIMA	MAE, RMSE, MAPE	–
24	[34]	9 years; Isfahan (Iran)	ANN, RF, SVM, KNN	Accuracy, MAE, RMSE, R	Accuracy = 90.1% (ANN)
25	[35]	Multi-station time series	GBM (MART), LSTM, DNN	RMSE, MAE, R	–
26	[36]	SENAMHI; 2019–2023	XGBoost, LightGBM	R^2^, RMSE, MAE	–

**Table 2:** Study area location table.

Node	Coordinates	Description	Representation
N1	−16.467415, −71.528841	Main square of Socabaya, characterized by high pedestrian and vehicular flow within the central urban area.	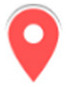
N2	−16.472396, −71.528795	Salaverry Avenue (primary corridor), a major traffic axis with frequent and continuous vehicle circulation.	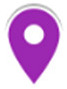
N3	−16.439499, −71.529540	Socabaya Avenue (primary corridor), a trunk avenue with sustained traffic demand.	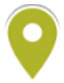
N4	−16.48898, −71.51013	Socabaya Penitentiary area, sector associated with a high concentration of heavy-duty transport vehicles.	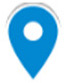

**Table 3:** Description of materials used in the IoT system.

Component	Description	Quantity	Purpose
Arduino Uno R3	Microcontroller board for electronic prototyping	1	Main board for local data processing
ESP32	Microcontroller with integrated WiFi and Bluetooth	1	IoT connectivity and data transmission
DHT11	Temperature and humidity sensor	1	Environmental monitoring
PMS5003	Air quality sensor (PM1.0, PM2.5, PM10)	1	Particulate matter monitoring
Cables and connectors	Interconnection accessories	Various	Electrical connections between components
Power supply	Power source for system components	1	Direct 5 V power supply
Breadboard	Temporary circuit assembly platform	1	Prototyping and testing

**Table 4:** Comparison of particulate matter measurements.

Source	PM2.5 Mean (µg/m^3^)	PM2.5 Range (µg/m^3^)	PM10 Mean (µg/m^3^)	PM10 Range (µg/m^3^)	Value Type
IoT Prototype	17.11	5.00–34.65	28.49 (8–58.44)	8.00–58.44	Local measurement
IQAir [26]	~28.5	-	-	-	Public reference
MINAM [2]	25 (annual)/50 (24 h)	-	50 (annual)/100 (24 h)	-	Regulatory limit

Note: IQAir values represent publicly available daily aggregates and are included solely as a contextual reference. Direct numerical comparison with IoT prototype readings should be interpreted with caution, as the two sources differ substantially in temporal resolution (10-min vs. daily aggregation), sensor technology, and spatial representativeness.

**Table 5:** Evaluated Algorithms and Hyperparameter.

N°	Algorithm	Key Hyperparameter	Avg. Runtime (s)	Computational Characteristics
1	SARIMAX [37]	order = (1, 1, 1), seasonal_order = (1, 1, 1, 24), exogenous variables enabled	278.25	Seasonal statistical model with exogenous variables
2	Linear Regression [38]	Default sklearn configuration	0.04	Linear parametric regression model
3	Ridge [39]	alpha = 1.0	0.01	L2-regularized linear regression model
4	Lasso [40]	alpha = 0.001, max_iter = 10,000	0.3	Sparse linear regression with feature selection
5	Elastic Net [41]	alpha = 0.001, l1_ratio = 0.5, max_iter = 10,000	0.29	Combined L1/L2 regularized regression model
6	kNN Regressor [42]	n_neighbors = 5, weights = distance	0.26	Distance-based instance learning model
7	Regression Tree [43]	max_depth = 10	0.06	Rule-based nonlinear decision model
8	Random Forest [44]	n_estimators = 200	12.19	Parallelizable tree-based ensemble method
9	Extra Trees [45]	n_estimators = 200	1.2	Highly randomized ensemble tree architecture
10	Gradient Boosting [46]	n_estimators = 300, learning_rate = 0.05, max_depth = 3, subsample = 0.8	4.26	Sequential boosting ensemble model
11	XGBoost [47]	n_estimators = 300, learning_rate = 0.05, max_depth = 6, subsample = 0.8, colsample_bytree = 0.8	0.55	Optimized gradient boosting framework
12	LightGBM [48]	n_estimators = 300, learning_rate = 0.05, num_leaves = 31, subsample = 0.8	0.44	Histogram-based efficient boosting model
13	CatBoost [49]	iterations = 300, learning_rate = 0.05, depth = 6	1.3	Ordered boosting architecture for robust learning
14	SVR (RBF) [50]	kernel = RBF, C = 10.0, epsilon = 0.1	12.22	Kernel-based regression requiring feature scaling
15	SGD Regressor [51]	alpha = 0.0001, learning_rate = optimal, eta0 = 0.001, max_iter = 2000	0.05	Incremental gradient-based linear optimization
16	MLP (Multilayer Perceptron) [52]	hidden_layer_sizes = (64, 32), max_iter = 1000	29.38	Feed-forward dense neural network
17	1D-CNN [53]	Conv1D filters = 64, kernel_size = 1, epochs = 50, batch_size = 16	71.5	Convolutional architecture for temporal pattern extraction
18	LSTM [54]	units = 64, Dense = 32, activation = tanh, epochs = 50, batch_size = 16	55.62	Recurrent neural network for long-term dependencies
19	GRU [55]	units = 64, Dense = 32, activation = tanh, epochs = 50, batch_size = 16	62.52	Reduced-parameter recurrent neural architecture
20	BiLSTM [56]	Bidirectional LSTM units = 64, Dense = 32	57.66	Bidirectional recurrent sequence modeling
21	Enc-Dec LSTM [57]	encoder_units = 64, decoder_units = 64	81.63	Sequence-to-sequence recurrent forecasting architecture
22	TCN (Temporal Convolutional Network) [58]	Conv1D filters = 64, dilation_rates = [1, 2], epochs = 50	68.46	Dilated causal convolutional temporal model
23	N-BEATS [59]	Dense layers = [128, 128, 64], epochs = 50	27.76	Deep fully connected forecasting architecture
24	Transformer [60]	num_heads = 2, Dense = 64, LayerNormalization epsilon = 1 × 10^−6^	74.9	Attention-based sequence modeling architecture
25	Informer/Autoformer [61]	Dense layers = [128, 64], Dropout = 0.2	13.92	Efficient transformer variant for long sequences
26	Quantile GBM (p50) [62]	objective = quantile, alpha = 0.5, n_estimators = 300	1.54	Probabilistic boosting-based quantile regression
27	NGBoost [63]	distribution = Normal, n_estimators = 400, learning_rate = 0.03	19.94	Probabilistic gradient boosting framework
28	Hybrid RF + MLP [64]	RandomForest n_estimators = 200, MLP hidden_layer_sizes = (64, 32)	13.62	Residual-learning hybrid ensemble model
29	KMeans + RF [65]	n_clusters = 3, RandomForest n_estimators = 150	8.94	Cluster-wise ensemble prediction framework
30	Naive [66]	No hyperparameters	0	Constant-time baseline forecasting model
31	Seasonal Naive [67]	season_length = 24	0	Seasonality-aware baseline statistical model
32	Moving Average [68]	window = 10	0	Low-cost smoothing-based forecasting approach
33	SES (Simple Exponential Smoothing) [69]	Default statsmodels configuration	0.04	Recursive exponential smoothing statistical method
34	Holt [70]	trend = additive	0.54	Trend-aware exponential smoothing model
35	Additive Holt–Winters [71]	trend = additive, seasonal = additive, seasonal_periods = 24	3.9	Seasonal smoothing model with additive decomposition
36	Multiplicative Holt–Winters [72]	trend = additive, seasonal = multiplicative, seasonal_periods = 24	3.49	Seasonal smoothing model with multiplicative decomposition
37	AR (Autoregressive) [73]	lags = 24	0.1	Lag-based autoregressive statistical model
38	MA (Moving Average) [74]	window = 3	0.2	Shock/error-based moving average model
39	ARMA [75]	p = 3, q = 3	11.54	Combined autoregressive and moving average model
40	ARIMA [76]	order = (1, 1, 1)	0.48	Differencing-based statistical forecasting model
41	SARIMA [77]	order = (1, 1, 1), seasonal_order = (1, 1, 1, 24)	97.7	Seasonal ARIMA forecasting architecture
42	DeepAR [78]	seq_len = 24, LSTM units = [128, 64], Dropout = 0.2, epochs = 40	404.35	Autoregressive probabilistic recurrent forecasting model
43	ARIMA–XGB Residual [79]	ARIMA = auto_arima, XGBoost n_estimators = 300, max_depth = 4	126.21	Hybrid statistical and boosting residual architecture
44	CNN–LSTM [80]	Conv1D filters = 64, kernel_size = 3, LSTM units = 128	279.81	Hybrid convolutional and recurrent temporal architecture

**Table 6:** Evaluated metrics.

Metric	Name	Formula	Interpretation
MAE	Mean Absolute Error	$MAE=\frac{\sum_{i=1}^{n}\left|y_{i}-x_{i}\right|}{n}$	The MAE is a measure of the mean size of prediction errors; all errors have the same contribution to the measure, there is no added weight on extreme deviations.
RMSE	Root Mean Squared Error	$\sqrt{1-\sum_{i=1}^{n}{\left(y_{i}-\hat{y_{i}}\right)}^{2}}$	The RMSE measures the quadratic mean of the prediction errors; higher values are assigned to big errors hence more susceptible to outliers.
R^2^	Coefficient of Determination	$1-\frac{\sum {\left(y_{i}-\hat{y_{i}}\right)}^{2}}{\sum {\left(y_{i}-\bar{y}\right)}^{2}}$	R^2^ gives the percentage of the variation in the observed data, which the model accounts; it shows the goodness of fit in general but does not measure the size of the error in prediction.
MAPE	Mean Absolute Percentage Error	$\frac{1}{n}\sum_{i=1}^{n}\left|\frac{y_{i}-\hat{y_{i}}}{y_{i}}\right|\times 100$	The MAPE is a statistical measure of the average relative error represented as a percentage; proportional deviations are focused on and the MAPE is therefore sensitive to small true values.

**Table 7:** PM2.5 evaluation metrics.

Model	MAE	RMSE	R^2^	MAPE	Time (s)
Last-Value Naive	6.63	8.21	−0.68	35.73	0.00
Seasonal Naive	7.54	9.39	−1.20	40.95	0.00
Moving Average	7.63	9.51	−1.26	38.71	0.00
SES	7.79	9.68	−1.34	39.40	0.04
Holt	7.87	9.76	−1.38	39.78	0.48
Holt–Winters Add	8.98	10.68	−1.84	46.85	3.40
Holt–Winters Mult	14.91	16.64	−5.91	85.39	3.48
AR	5.38	6.28	0.01	40.22	0.10
MA	8.54	10.38	−1.79	43.80	0.21
ARMA	5.30	6.22	0.03	39.72	10.13
ARIMA	7.79	9.68	−1.34	39.42	0.41
SARIMA	8.99	10.68	−1.85	46.87	88.66
SARIMAX	2.77	3.44	0.71	16.97	288.50
Linear Regression	2.92	3.52	0.69	18.32	0.04
Ridge	2.92	3.52	0.69	18.32	0.01
Lasso	2.92	3.52	0.69	18.32	0.26
Elastic Net	2.92	3.52	0.69	18.34	0.25
kNN Regressor	1.95	2.49	0.85	13.56	0.16
Regression Tree	1.65	2.08	0.89	11.69	0.05
Random Forest	1.72	2.16	0.88	12.23	13.65
Extra Trees	1.88	2.37	0.86	13.38	1.37
Gradient Boosting	1.58	1.99	0.90	11.23	4.47
XGBoost	1.60	2.00	0.90	11.34	0.65
LightGBM	1.59	1.99	0.90	11.26	0.49
CatBoost	1.58	1.99	0.90	11.22	1.45
SVR (RBF)	1.88	2.36	0.86	12.81	13.45
SGD Regressor	2.93	3.55	0.69	18.04	0.04
MLP	1.95	2.47	0.85	12.93	12.43
1D-CNN	1.82	2.30	0.87	12.41	68.85
LSTM	1.83	2.31	0.87	12.59	48.33
GRU	1.82	2.30	0.87	12.49	61.67
BiLSTM	1.79	2.27	0.87	12.39	68.33
Enc-Dec LSTM	1.71	2.18	0.88	12.19	80.22
TCN	1.74	2.20	0.88	11.99	60.71
N-BEATS	1.80	2.29	0.87	12.42	27.83
Transformer	1.83	2.31	0.87	12.52	64.73
Informer–Autoformer	2.77	3.51	0.69	16.28	14.19
Quantile GBM (p50)	1.58	1.99	0.90	11.26	2.82
NGBoost	1.58	1.99	0.90	11.23	19.52
DeepAR	1.74	2.23	0.88	12.22	366.40
ARIMA-XGB Residual	15.20	16.61	−5.88	88.11	99.32
CNN-LSTM	1.71	2.16	0.88	12.19	255.13
Hybrid RF + MLP	1.72	2.16	0.88	12.18	13.60
KMeans-RF	1.73	2.17	0.88	12.28	9.11

**Table 8:** PM10 evaluation metrics.

Model	MAE	RMSE	R^2^	MAPE	Time (s)
Last-Value Naive	10.45	12.68	−0.47	34.94	0.00
Seasonal Naive	11.44	14.36	−0.89	36.62	0.00
Moving Average	12.16	15.20	−1.12	36.83	0.00
SES	10.98	13.57	−0.69	35.01	0.04
Holt	10.86	13.38	0.64	34.92	0.60
Holt–Winters Add	12.23	14.80	−1.01	38.34	4.40
Holt–Winters Mult	35.00	42.32	−15.42	155.20	3.50
AR	9.04	10.39	0.01	39.38	0.10
MA	10.93	13.81	−0.86	34.14	0.20
ARMA	8.93	10.31	0.03	38.92	12.95
ARIMA	10.95	13.53	−0.68	34.99	0.54
SARIMA	12.23	14.82	−1.01	38.35	106.75
SARIMAX	4.45	5.50	0.72	18.14	268.00
Linear Regression	4.77	5.70	0.70	17.30	0.03
Ridge	4.77	5.70	0.70	17.31	0.01
Lasso	4.77	5.75	0.70	17.31	0.33
Elastic Net	4.77	5.75	0.70	17.32	0.33
kNN Regressor	3.00	3.88	0.86	12.15	0.37
Regression Tree	2.50	3.16	0.91	10.30	0.06
Random Forest	2.62	3.30	0.90	10.94	10.73
Extra Trees	2.87	3.60	0.88	12.09	1.04
Gradient Boosting	2.39	3.01	0.92	9.89	4.04
XGBoost	2.41	3.04	0.92	9.98	0.44
LightGBM	2.41	3.04	0.92	9.95	0.39
CatBoost	2.39	3.01	0.92	9.90	1.14
SVR (RBF)	2.87	3.66	0.88	11.38	10.98
SGD Regressor	4.86	5.80	0.69	17.50	0.06
MLP	2.58	3.28	0.90	10.60	46.33
1D-CNN	2.60	3.34	0.90	10.46	74.15
LSTM	2.76	3.54	0.89	11.03	62.91
GRU	2.72	3.46	0.89	10.87	63.37
BiLSTM	2.81	3.60	0.88	11.34	46.98
Enc-Dec LSTM	2.53	3.22	0.91	10.35	83.05
TCN	2.55	3.25	0.90	10.34	76.20
N-BEATS	2.70	3.46	0.89	11.26	27.69
Transformer	3.36	4.24	0.84	12.78	85.08
Informer–Autoformer	4.05	5.24	0.75	14.13	13.64
Quantile GBM (p50)	2.39	3.01	0.92	9.89	0.26
NGBoost	2.39	3.01	0.92	9.89	20.36
DeepAR	2.58	3.31	0.90	10.47	442.30
ARIMA-XGB Residual	8.42	9.73	0.13	36.66	153.10
CNN-LSTM	2.57	3.29	0.90	10.52	304.49
Hybrid RF + MLP	2.61	3.29	0.90	11.03	13.64
KMeans-RF	2.63	3.32	0.90	10.97	8.78

**Table 9:** Extended Forecast Horizon (t + 6/60 min ahead) Results.

Model	PM2.5 R^2^	PM10 R^2^	Avg Runtime (s)
Regression Tree	0.8921	0.8948	0.08
CatBoost	0.9018	0.9082	1.59
NGBoost	0.9016	0.9095	27.81
N-BEATS	0.8860	0.8851	81.63
Hybrid RF + MLP	0.8920	0.9065	20.56
Stacking Ridge K-Fold	0.9010	0.9098	465.13

**Table 10:** Comparison of the performance of ensemble methods of various datasets.

Ensemble	R^2^ Arequipa (PM2.5)	R^2^ Mexico (PM2.5)	R^2^ Arequipa (PM10)	R^2^ Mexico (PM10)
Weighted Ensemble	0.9005	0.4611	0.9158	0.4605
Stacking Ridge	0.8941	−0.1119	0.9099	−0.1589
Stacking Ridge K-Fold	0.9019	0.5535	0.917	0.5582

**Table 11:** Framework generalization analysis.

Aspect	Arequipa Dataset	Mexico Dataset
Quality of data	High	Medium
Average R^2^ (ensemble)	~0.91	~0.55
Best ensemble method	K-Fold	K-Fold
Stability of models	High	Moderate
Sensitivity to Distribution Shift	Low	High

**Table 12:** Direct reference to the suggested framework and the exemplary strategies of the literature.

Study	IoT Deployed	N° Algorithms	Homogeneous Conditions	Ensemble/Stacking	Medium- Altitude Context
Zhang et al. [22] (China)	No (official station)	2	Partial	Hybrid RF-LSTM	No
Banciu et al. [15] (Rumania)	Yes	3	Partial	LSTM-GRU	No
Joharestani et al. [29] (Irán)	No (satellite)	3	Yes	No	No
Liu et al. [11] (China)	No (station)	4–6	Partial	Hybrid DL	No
Present Study (Arequipa)	Yes (4 nodes)	44	Yes (Complete)	Stacking Ridge K-Fold	Yes (2335 m.a.s.l.)

## Data Availability

The data presented in this study are available on request from the corresponding author.
